# Label-free fluorescence lifetime imaging can distinguish cancer from healthy tissue in spontaneously occurring canine oral tumors

**DOI:** 10.1038/s41598-026-37001-3

**Published:** 2026-01-23

**Authors:** Stephanie Goldschmidt, Laura Marcu, Katjana Ehrlich, Mohamed Abul Hassan, Iris Rivas, Andrew Birkeland, Xiangnan Zhou, Julien Bec, Alba Alfonso Garcia, Shuai Chen, Yichu Chen, Yash Tipirneni, Max Kampe, Abigail Weir, Abraham Morales, Christine Ly, Robert Rebhun, Brian G. Murphy, Natalia Vapniarsky

**Affiliations:** 1 Department of Surgical & Radiological Sciences, School of Veterinary Medicine, University of California-Davis, Davis, CA USA; 2 Department of Biomedical Engineering, University of California-Davis, Davis, CA USA; 3 Department of Otolaryngology Head and Neck Surgery, School of Medicine, University of California-Davis, Davis, CA USA; 4 Division of Biostatistics, Department of Public Health Sciences, University of California-Davis, Davis, CA USA; 5 Department of Pathology, Microbiology and Immunology, School of Veterinary Medicine, University of California-Davis, Davis, CA USA; 6 Department of Neurological Surgery, University Of California-Davis, Davis, United States

**Keywords:** Squamous cell carcinoma, Oral tumors, Fluorescence guided surgery, Fluorescence lifetime imaging, 5-aminovelic acid, Protoporphyrin IX, Biological techniques, Biomarkers, Cancer, Oncology

## Abstract

Post-surgical local recurrence of oral cancer remains unacceptably high across species due to the lack of non-invasive tools capable of accurately delineating tumor from healthy tissue. Label-free fluorescence lifetime imaging (FLIm) has shown moderate-to-high success in human head and neck squamous cell carcinoma, but it is unclear whether diagnostic accuracy can be enhanced by incorporating exogenous fluorophores that selectively accumulate in cancer. This study evaluated the performance of 5-ALA induced Protoporphyrin IX (PpIX) fluorescence and autofluorescence FLIm features to discriminate epithelial cancer and healthy tissues in a spontaneous large animal model of disease (15 pet dogs). Fluorescence emission parameters (e.g. lifetimes, intensity ratios, phasors and Laguerre coefficients) differed significantly (*p* < 0.001) between cancer and healthy tissues in both autofluorescence and PpIX channels. However, autofluorescence features, particularly lifetimes in Channel 1 (390 nm, collagen-sensitive) and intensity ratios in Channel 2 (470 nm, NADH-sensitive), provided the strongest in vivo discrimination. These results demonstrate that label-free FLIm alone is sufficient to distinguish epithelial oral cancers from healthy tissue in dogs, and that the addition of exogenous markers such as 5-ALA–induced PpIX, does not markedly improve diagnostic accuracy enough to warrant incorporation into flourescence imaging approaches.

**Supplementary Information:**

The online version contains supplementary material available at 10.1038/s41598-026-37001-3.

## Introduction

To date, there is a lack of non-invasive technology capable of accurately delineating oral neoplastic from normal tissues in vivo*.* Fresh frozen histopathology is standard for human head and neck squamous cell carcinoma (HNSCC) to determine margin status and guide decision making while still in the operating room. However, intraoperative frozen sections have inherent inaccuracies, with one study reporting a sensitivity of only 10.8% for accurately defining margin status from the tumor bed^1^. Although the majority of research supports a lower false negative rate for fresh-frozen sampling, closer to 30%, with significantly superior outcomes using specimen-oriented frozen analysis,^2^ this technology is only applicable to soft tissue margins and is largely dependent on the surgeon properly orienting the specimen to guide additional surgical resection in cases of incomplete margins^3,4^.

As there is no reliable pre- or intra-operative diagnostic technique, a surgical safety margin is required to ensure the removal of all potential non-visible neoplastic cells. Yet, in oral cancer, excision of bordering benign tissue typically results in substantial structural, functional, and cosmetic side effects. Most notably, changes in the ability to prehend, chew, and vocalize. As the use of surgical safety margins (ideally 10 mm gross margins) is limited by function, local recurrence rates remain unacceptably high in this region. In fact, HNSCC has consistently remained the 3rd most common solid tumor with positive surgical margins (PSM) in humans^5^. Similar pitfalls are documented in companion dogs with local recurrence rates reported up to 28% for oral squamous cell carcinoma (SCC)^6^. It remains unclear if local recurrence is due to persistent neoplastic cells or presence of field cancerization^7^, which was not accurately identified and treated. Conversely, in other epithelial tumors, specifically ameloblastoma, recurrence rates remain low despite narrow margins, suggesting that the current surgical approaches may be overly aggressive and more normal tissue could be spared^8^. Collectively, there is an unmet need to identify non-invasive intraoperative technologies that can accurately and reliably delineate neoplastic from healthy epithelial tissue in the oral cavity to improve patient outcomes across species.

Fluorescence spectroscopy and imaging shows promise for bridging this diagnostic gap in both humans and dogs^9–18^. In brief, changes in fluorescence emission and related spectral properties, either from cancer-driven changes in tissue autofluorescence or administration of an exogenous fluorophore that preferentially accumulates in cancer, can give real-time visual intraoperative surgical guidance. Endogenous fluorescence in the oral cavity is primarily driven by the intrinsic tissue fluorophores collagen, Nicotinamide Adenine Phosphate Dinucleotide (NAD(P)H), and Flavin Adenine Dinucleotide (FAD)^19^. Collectively, these molecules have altered spectral properties in cancer secondary to decreased collagen crosslinking^20^ and metabolic shifts including decreased use of oxidative phosphorylation (Warburg effect) and a more acidic environment. ^21–23^ Utilization of endogenous fluorescence offers the advantage of avoiding potential side effects associated with externally administered substances. Yet, achieving high tissue discrimination can be more challenging when focusing solely on endogenous tissue properties, especially when relying only on visual fluorescence intensity seen with the naked eye. Thus, exogenous fluorophores are often employed to increase the signal-to-noise ratio of the neoplasia compared to normal background tissue. Non-specific exogenous fluorophores have increased clinical applicability compared to cancer-specific fluorophores, as they are readily available, relatively inexpensive, and can be used across a variety of tumor types and heterogeneities, regardless of whether they harbor a particular mutation or overexpress a surface protein. 5-aminolevulinic acid (5-ALA) is a naturally occurring pro-drug that is metabolized to the active fluorophore, protoporphyrin IX (PpIX) via the heme biosynthetic pathway^17^. PpIX, the active fluorophore, preferentially accumulates in neoplastic tissue when 5-ALA is given in excess, although the exact mechanism of action remains unknown^24,25^.

5-ALA induced PpIX fluorescence has been reported to have a high sensitivity and low specificity of 89.5% and 50%, respectively for visually identifying non-oral neoplasms in dogs^12^. A recent systematic review in human patients identified 13 studies (*n* = 397 patients) that utilized 5-ALA to diagnose HNSCC, reporting 5 studies with a sensitivity of 100% and 8 studies with sensitivity ranging from 82.7%-94.6%.^26^ However, neither canine nor human studies report on the ability to accurately identify the tumor margin or presence of residual disease with this technique, which will inherently be affected by low cellularity. In non-oral sites (e.g. glioma resection), use of 5-ALA induced PpIX fluorescence to guide surgical decision making has poor accuracy in cancers with low cellularity (poor fluorescence emission) or high tissue autofluorescence, limiting its clinical use. Yet, use of fluorescence lifetime imaging (FLIm) has shown improved sensitivity in the face of low tumor background ratio (TBR) caused by poor cellularity^27^. FLIm quantifies changes in the lifetime (time of degradation) of the emitted fluorescence signal. Thus, it is a quanitative, not subjective measurement and is thus less affected by TBR and system-specific imaging parameters^28^. Capitalizing on fluorescence lifetimes and related spectral properties can potentially be leveraged to increase the accuracy of 5-ALA induced PpIX fluorescence and facilitate clinical translation of this technique among oral tumors with varying cellular densities.

Binary diagnosis of cancer versus healthy tissue in human HNSCC has utilized autofluorescence FLIm^29,30^. This technique was able to discriminate healthy tissue from cancer with an area under the curve (AUC) of 0.88^29^ and detect positive surgical margins with an AUC of 0.74.^31^ It has not been determined if diagnostic accuracy of FLIm can be further improved with the addition of an exogenous fluorophore, such as 5-ALA induced PpIX, which is known to accumulate in epithelial cancers across species. The goal of this study was to evaluate both autofluorescence and PpIX fluorescence decay characteristics simultaneously via FLIm in a spontaneous large animal model of disease (pet dogs) to evaluate that ability to distinguish between oral epithelial cancer and healthy tissue in real-time during a surgical procedure. We first validated the ability to 5-ALA to induce PpIX visual fluorescence in canine epithelial oral cancer cell lines *in vitro.* We then proceeded to determine clinical accuracy of simultaneous autofluorescence and PpIX FLIm collected both in vivo prior to tumor resection and ex vivo immediately post-resection to differentiate oral epithelial neoplasia from healthy tissue.

## Results

### 5-ALA induced PpIX in canine oral cancer cell lines

**Fig. 1:**
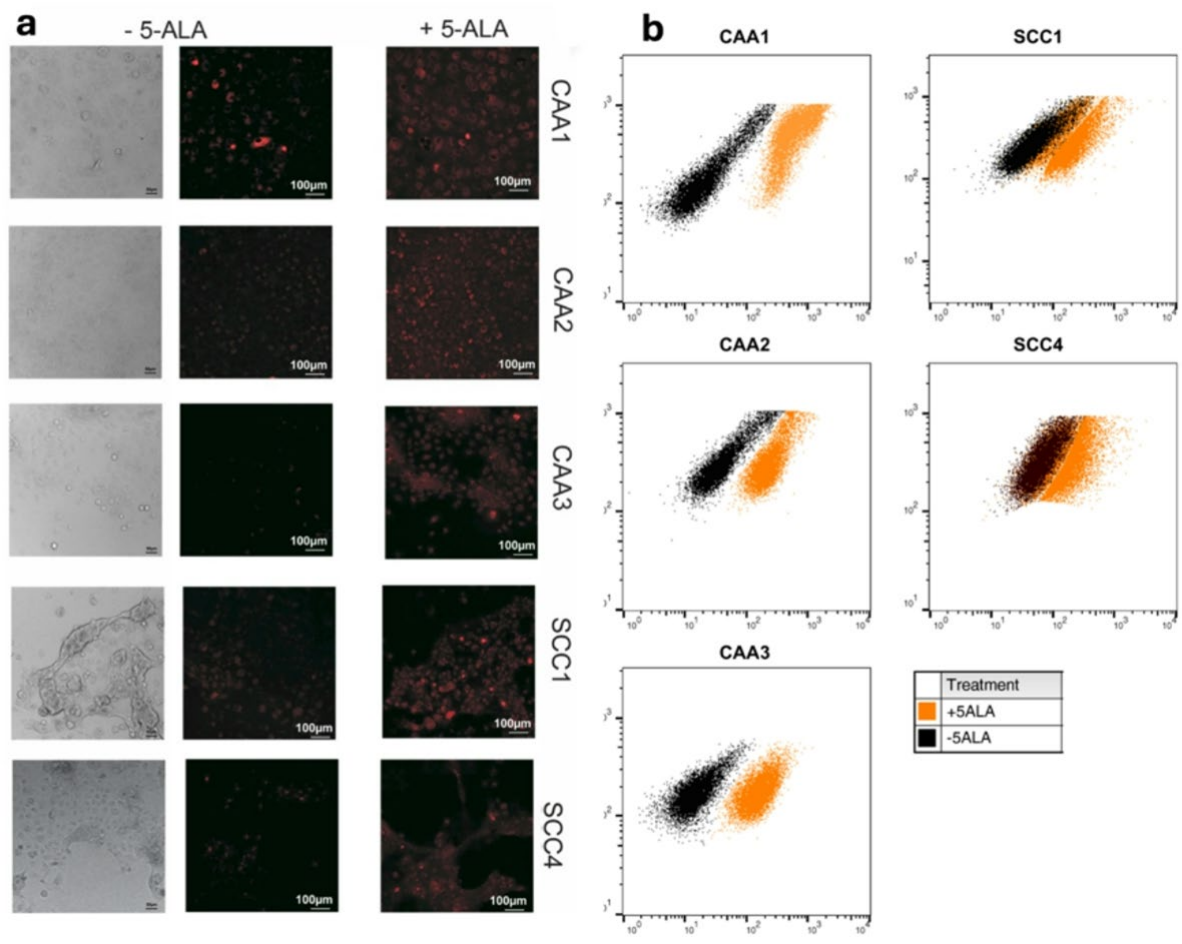
PpIX accumulation in Canine Epithelial Oral Cancer Cell Lines. (**a**) Confocal microscopy of canine CAA and SCC cell lines with and without incubation with 5-ALA revealing increased mean florescence intensity under excitation at 470 nm when incubated with 5-ALA (+ 5-ALA) compared to the control (-5-ALA, cell autofluorescence only). (**b**): Flow cytometry of canine cell lines with and without 5-ALA incubation showing increased percent of fluorescent cells captured in channel 3, although minimal change was noted in SCC4 compared to other cell lines.

There was an increase in percentage of fluorescent cells in the gate defined as positive after 5-ALA exposure in all cell lines on flow cytometry. The highest percentage of positive cells post 5-ALA exposure was observed in canine acanthamatous ameloblastoma (CAA) with CAA1-3 showing 94%, 92%, and 96%, respectively. Combined data from all cell lines with and without 5-ALA revealed a significant (*p* = 0.005) increase in mean fluorescence intensity (FI) in the FL3 channel. These findings were confirmed with increased mean FI in 5-ALA incubated cells compared to the control (autofluorescence) on confocal microscopy (Fig. 1).

### Animals and mechanistic action of 5-ALA induced PpIX accumulation

Following validation of PpIX fluorescence on representative canine epithelial oral cancer cell lines, spectral data were acquired from 11 dogs with oral SCC and 4 dogs with CAA (Table 1). 5-ALA was given at a median of 190 (170–254) mins prior to imaging. No dogs had any side effects related to the 5-ALA oral administration. All dogs with gross disease had visual fluorescence seen with the naked eye of cancer under blue light excitation (405 nm). This was confirmed by pathology of the center of the lesion where fluorescence was the most prominent compared to non-fluorescence regions which were pathologically confirmed as normal tissue. Two dogs exhibited visual fluorescence of pathologically confirmed non-cancerous tissue (inflammatory scar tissue from an excisional biopsy and a viral papilloma).

**Table 1:** Patients prospectively recruited into the clinical trial. Patient specific information including breed, sex (male castrated (MC) or female spayed (FS)), and age (years old (yo)) is reported. Tumor variant as a conventional/papillary squamous cell carcinoma (SCC) or solid/canine acanthamatous ameloblastoma (CAA) is reported. Tumor location and size is reported using the WHO TNM staging system as T1: <2 cm, T2: 2–4 cm, T3 > 4 cm. Presence of locoregional metastasis and staging methodology is reported. Within this study Sentinel lymph node (SLN) mapping was performed by indirect computed tomography lymphangiography. Lastly, presence of distant metastasis and staging methodology with radiology or computed tomography (CT) is reported.

Patient Number	Dog Signalment	Variant	Tumor size	Tumor Location	Locoregional metastasis, staging performed	Distant Metastasis, staging performed	Time from 5-ALA to imaging	Visual PpIX fluorescence?
Squamous Cell Carcinoma								
9	6 yo MC Siberian Husky	Conventional SCC	T1	Rostral mandible	No, histology SLN	No, thoracic CT	213 min	Yes
11	8 yo FS Labrador Retriever Mix	Conventional SCC	T2	Rostral mandible	No, histology SLN	No, thoracic CT	209 min	Yes
4	2 yo MC Australian Shepherd	Papillary SCC transformed from viral papilloma	T3	Caudal mandible	Unknown, owner declined screening	No, thoracic CT	225 min	Yes, papilloma at distal end of tumor also fluoresced
14	3 yo MC Golden Retriever	Papillary SCC	T1	Rostral maxilla	No, cytology bilateral Mandibular LN	No, thoracic CT	200 min	Yes
17	9 yo MC Miniature Poodle	Conventional SCC	Scar revision, T1 prior to excisional biopsy by rdvm. Final pathology: no cancer	Caudal maxilla/ cheek pouch	No, histology of SLN	No, thoracic CT	185 min	Yes, near inflammation caudally only in region of previous surgery
18	7 yo MC Labrador Retriever	Conventional SCC	Scar Revision, T1 prior to excisional biopsy at VMTH, no sutures. Final pathology: no cancer	Caudal mandible	No, histology of SLN	No, thoracic CT	224 min	No
20	10 yo FS Chinese Crested	Conventional SCC	T2	Caudal mandible	No, histology of SLN	No, thoracic CT	190 min	Yes
6	13 yo FS Maltese	Conventional SCC	T3	Rostral-caudal mandible	No, histology from complete lymph node dissection (CLND)	No, thoracic CT	185 min	Yes
23	12 yo MC Boxer Mix	Conventional SCC	T3	Rostral mandible	No, histology from CLND	No, thoracic CT	180 min	Yes
15	10 yo MC Pit Bull	Conventional SCC	T2	Rostral maxilla	Unknown, owner declined screening	Unknown, owner declined screening	189 min	Yes
29	13 yo MC Shih Tzu	Conventional SCC	T3	Caudal Mandible	Yes, histology from CLND	No, thoracic CT	180 min	Yes, at periphery of tumor only
Ameloblastoma								
16	10 yo FS American Eskimo	CAA	T2	Rostral mandible	No, histology SLN *incisional biopsy looked more consistent with SCC, final was CAA	No, thoracic radiographs	254 min	Yes
2	9 yo FS English Bulldog	CAA	T1	Rostral maxilla	Unknown, owner declined screening	Unknown, owner declined screening	212 min	No * given in pill
22	12 yo FS German Shepherd Dog	CAA	T2	Caudal maxilla	Unknown, owner declined screening	Unknown, owner declined screening	170 min	Yes
27	4 yo MC Cocker Spaniel Mix	CAA	T2	Caudal Maxilla	Unknown, owner declined screening	No, thoracic CT	185 min	Yes

**Fig. 2:**
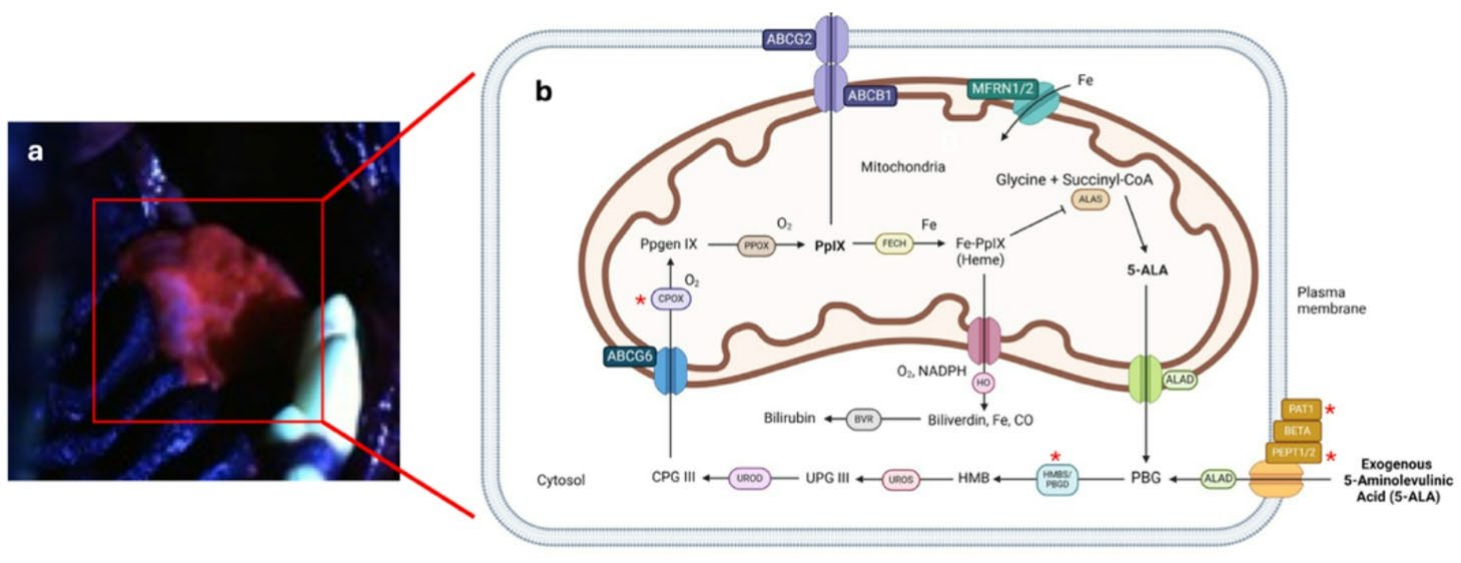
Protoporphyrin IX (PpIX) accumulation in canine oral squamous cell carcinoma after exogenous 5-aminolevulinic acid (5-ALA) administration. (**a**) Visual fluorescence (pink) of 5-ALA induced PpIX under blue light stimulation (450 nm) of a canine oral tumor on the hard palate that preferentially accumulated PpIX compared to the surrounding normal tissue. (**b**) Heme biosynthetic pathway illustrating the enzymes involved in breakdown of 5-ALA to PpIX, then heme and bilirubin. * Represents enzymes that were upregulated in fluorescent canine oral squamous cell carcinoma compared to non-fluorescent normal oral mucosa.

Eight patients with oral SCC had tissue analyzed for differing gene expression utilizing real time PCR in regions of visual fluorescence compared to non-fluorescent normal tissue. In the patient with papilloma induced carcinoma (patient 4), tumor tissue that fluoresced (4a) and a distant papilloma that fluoresced (4b) were both analyzed. Of note, the tumor surface contained multiple active papillomas. When the data from the papilloma case is excluded, HMBS, CPOX, and PEPT 2 (SLC15A2) were upregulated in all cases. Further, ALAS1 and PAT 1(SLC6A13) were upregulated in 6/7 patients (Supplemental Table 1). Taken together, visual fluorescence in oral SCC occurred due to preferential uptake of 5-ALA into the cell mitochondria and increased production of mitochondrial PpIX from intracellular 5-ALA (Fig. 2, *upregulated enzymes in the 5-ALA metabolic pathway in fluorescent SCC tissue compared to non-fluorescent normal tissue).

### Fluorescent lifetime imaging

**Fig. 3:**
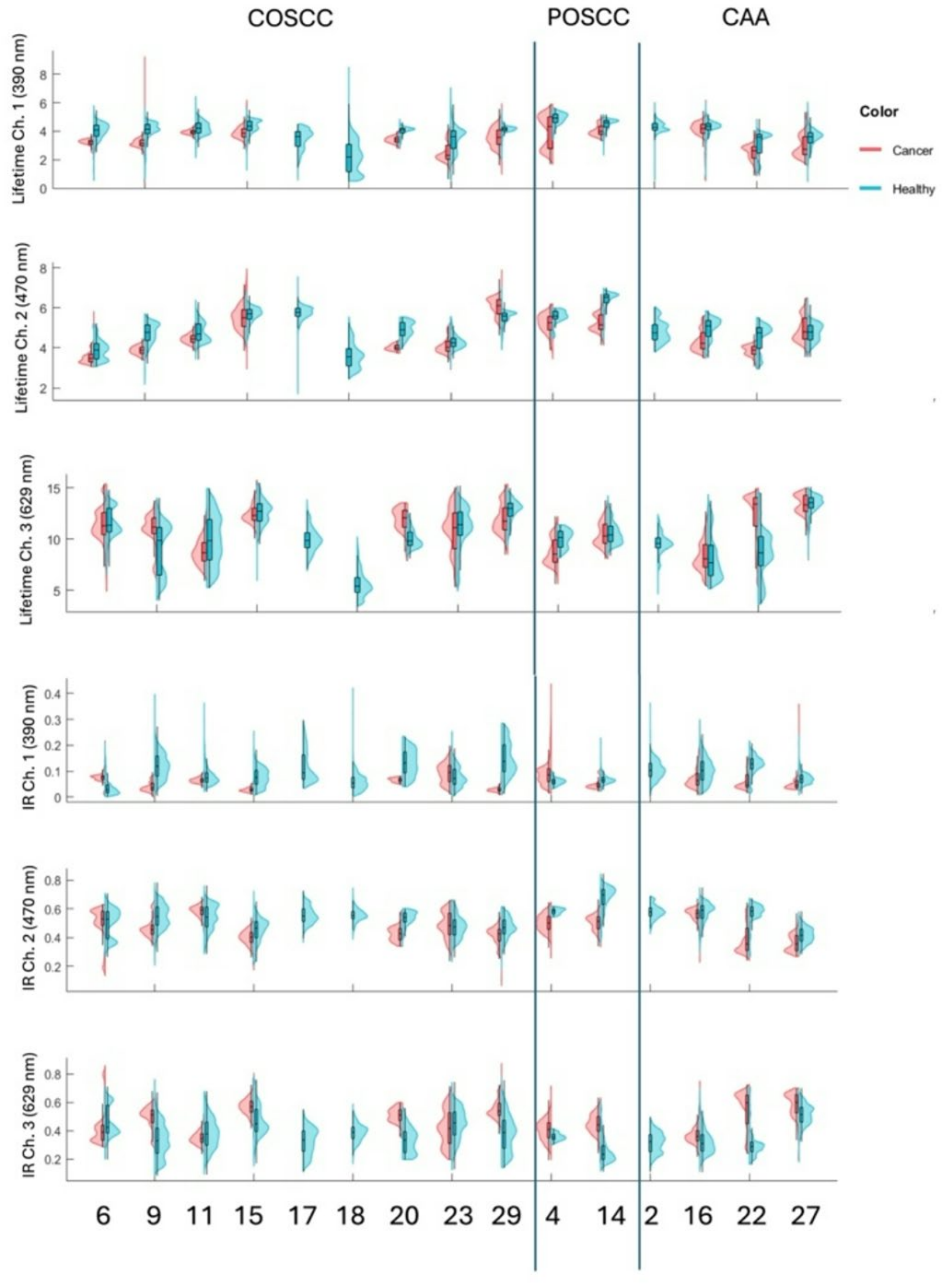
All included in vivo fluorescence lifetimes and intensity ratios of epithelial oral tumors in the autofluorescence and exogenous (PpIX) spectral channels across all patients. Patient number is shown on the X axis, and patients are grouped by tumor type to visually asses for trends.

A total of 532,343 total FLIm data points were collected across the 15 patients. After initial data clearing for points that did not have pathology labels as well as gain and signal to noise ratio (SNR) filtering, there were 110,283 in vivo points and 106,888 ex vivo points across all patients (Supplemental Fig. 1). For each data point, fluorescence decays were characterized across the 3 spectral channels associated with the emission bands of Collagen (channel 1: 390/40 nm), NADPH (channel 2: 470/28 nm), and PpIX (channel 3: 629/53 nm) generating multiple spectral features per FLIm point including 3 average Lifetimes (1 per channel), 3 intensity ratios (IR; 1 per channel), 36 Laguerre coefficients (12 per channel), and 12 lifetime phasors (4 per channel). A violin plot showing healthy and cancer data points (in vivo lifetimes and IR across the 3 channels) per patient included in analysis are shown in Fig. 3.

### In vivo tissue discrimination

**Fig. 4:**
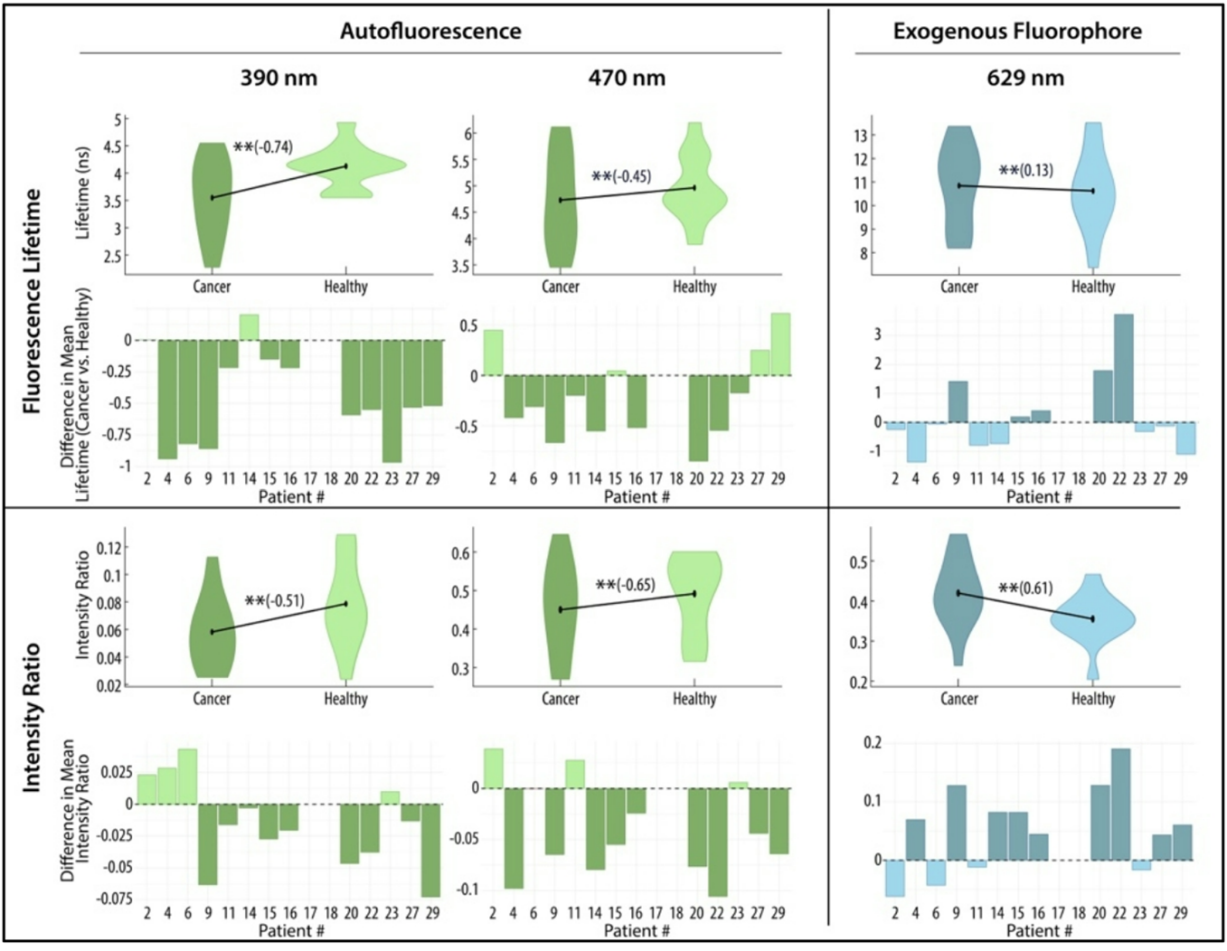
In vivo fluorescence lifetimes and intensity ratios of for all patients. Violin plots show the lifetimes and intensity ratios of cancer and healthy tissues aggregated from all patients (*n* = 15). ** denotes *p* < 0.001 on univariable analysis. The effect size of each FLIm parameter to discriminate between cancer and healthy tissue is depicted in parenthesis. Below the violin plots, the qualitative difference between cancer and healthy mean lifetimes and intensity ratios, respectively, per patient is shown. Negative values mean the spectral feature was lower in cancer, while positive values mean the spectral feature was higher in cancer for that patient.

In vivo data points were aggregated across all patients to globally assess tissue discrimination in canine oral epithelial tissues. FLIm parameters were significantly different (*p* < 0.001) in their means between aggregated cancer and healthy data points across all channels based on univariable analysis with weighted linear mixed-effect models accounting for within animal correlation (Supplementary Table 2). To explore the discriminatory power of each feature in the univariate model to discriminate cancer from healthy data points, effect size was calculated with effect of ± 0.2 considered small, ± 0.5 considered medium, and ± 0.8 large. The features with the largest discriminatory power between tissue types were in the autofluorescence channels, included Channel 1 (390 nm) lifetime with an effect size of -0.74 and Channel 2 (490 nm) lifetime and phasors with an effect size of -0.65 and − 0.80, respectively. Although the intensity ratio of the PpIX channel (629 nm) had good discrimination between tissue types (effect size of 0.61), all other FLIm parameters in this channel had a very small effect. The effect size for each imaging feature to discriminate between cancer and healthy data points across all patients is shown in parenthesis next to the violin plot of all data points (Fig. 4).

To visually appreciate the trends between healthy and cancer data points on a patient specific level and understand the repeatability of each spectral feature for tissue discrimination a bar graph is shown (difference in mean lifetime or IR between health and cancer per patient). It can be seen that Channel 1 lifetimes were the most predictable features, with lower mean lifetimes in all but 1 patient (Fig. 4).

Multivariable analysis was then conducted using weighted logistic mixed-effects models. The initial model included intensity ratio, lifetime, and principal components of the multiple phasors and Laguerre coefficients. Specifically, due to the multiple phasor and Laguerre coefficients per channel, multi-leveled weighted principal component analysis (PCA) was performed to reduce data dimension and multicollinearity. The first two principal components were used in multivariable analysis, which explained about 77% to 98% of variability (Supplementary Table 3).

The model was further refined through a backward stepwise selection, removing one predictor at a time based on p-values (removing those with *p* > 0.05) and high variance inflation factors (to reduce collinearity). In the multivariable model, only features from the autofluorescence channels were maintained as PpIX lifetimes and intensity ratios had high variance inflation factors (VIF = 207 and 42, respectively), where VIF > 10 indicates serious multicollinearity. This suggested that they are strongly correlated with other FLIm parameters, indicating redundancy in predictive information for differentiating tissue types. In the final model, lower lifetimes and intensity ratios in the autofluorescence channels predicted cancer, with the channel 1 lifetime having the strongest association between a FLIm feature (predictor) and tissue type (Table 2).

**Table 2:** Summary of coefficient estimates from weighted logistic mixed-effects model using the in vivo data for predicting tissue type with FLIM parameters and principal components (PCs). Coefficient > 0 means that the higher value of the predictor, the higher probability that it is cancer; coefficient < 0 means that the higher value of the predictor, the lower probability that it is cancer; the magnitude of coefficient indicates the association between the predictor and tissue type (the larger, the stronger).

Predictor	Channel	Principal component	Coefficient (SE)	*P*-value
Intensity ratio	1 (390 nm)	-	-1.123 (0.017)	< 0.001
	2 (470 nm)	-	-1.798 (0.023)	< 0.001
Lifetime average	1 (390 nm)	-	-1.816 (0.031)	< 0.001
	2 (470 nm)	-	-0.709 (0.033)	< 0.001
Phasor (Between-Subject PC)	1 (390 nm)	PC1	-1.619 (0.289)	< 0.001
Phasor (Within-Subject PC)	1 (390 nm)	PC1	-0.048 (0.009)	< 0.001
		PC2	-0.156 (0.006)	< 0.001
	3 (629 nm)	PC1	-0.249 (0.008)	< 0.001
		PC2	0.350 (0.011)	< 0.001
Laguerre coefficient (Within-Subject PC)	1 (390 nm)	PC1	0.111 (0.004)	< 0.001
		PC2	-0.729 (0.009)	< 0.001
	2 (470 nm)	PC1	-0.069 (0.008)	< 0.001
		PC2	0.078 (0.005)	< 0.001
	3 (629 nm)	PC1	-0.509 (0.008)	< 0.001
		PC2	0.221 (0.006)	< 0.001

**Fig. 5:**
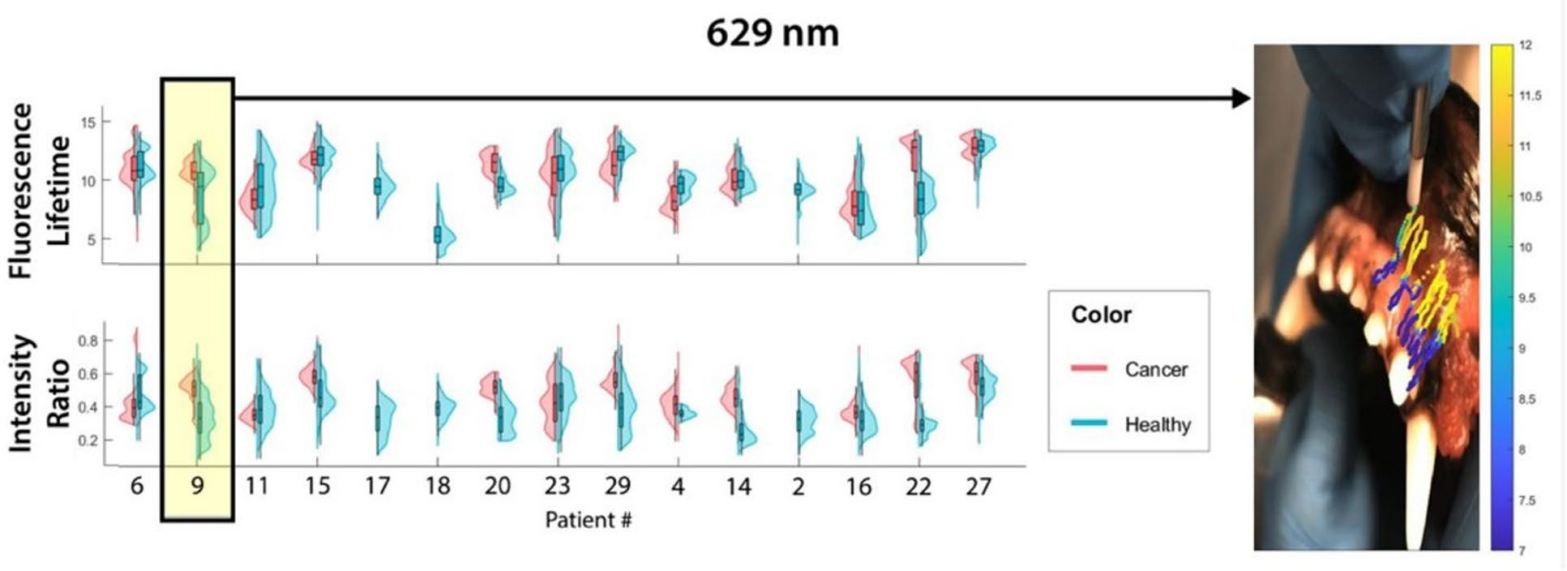
Example of a patient that had a large range of Channel 3 fluorescence lifetimes and intensity ratios in healthy tissue. An overlay of the lifetimes (ns shown by the color bar) obtained in this patient’s scan show the distinctly lower lifetime in the gingival tissue compared to the buccal mucosa despite no obvious visual PpIX fluorescence to the naked eye in either region.

The poor ability of Channel 3 (629 nm) spectral features to predict cancer may be explained by evaluation of the raw data per patient which shows a very high range of lifetimes in healthy tissue. To further explore this finding, regions of interest were outlined in patient healthy tissue scans to identify if any specific tissue type or anatomy was primarily driving changes. It was found that even in the absence of visual PpIX fluorescence seen with the naked eye and/or gingival inflammation, gingiva and mucosa had distinctly different lifetimes in the 629 nm channel (Fig. 5).

### Ex vivo tissue discrimination

**Fig. 6:**
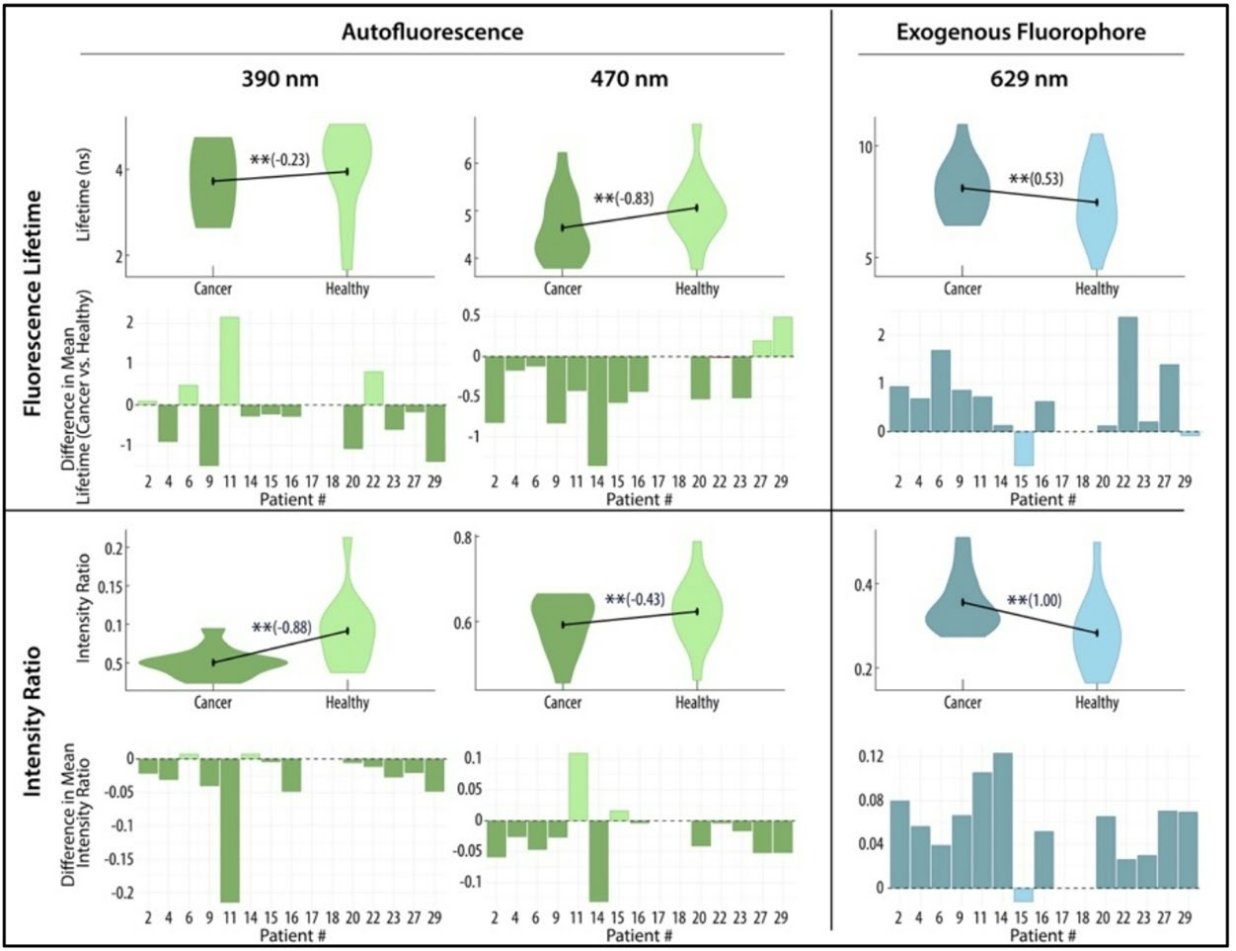
Ex vivo fluorescence lifetimes and intensity ratios of epithelial oral tumors in the autofluorescence and exogenous (PpIX) spectral channels in all patients. Violin plots show the lifetimes and intensity ratios of cancer and healthy tissues aggregated from all patients (*n* = 15). ** denotes *p* < 0.001 on univariable analysis. The effect size of each FLIm parameter to discriminate between cancer and healthy tissue is depicted in parenthesis. Below the violin plots, the qualitative difference between cancer and healthy mean lifetimes and intensity ratios, respectively, per patient is shown. Negative values mean the spectral feature was lower in cancer, while positive values mean the spectral feature was higher in cancer for that patient.

Ex vivo spectral data points were analyzed the same as the in vivo data starting initially with univariable analysis and then through a logistic mixed-effects model. It was found that *ex-vivo* FLIm parameters were significantly different (*p* < 0.001) in their means between aggregated cancer and healthy data points across all channels based on univariable analysis (Supplementary Table 4). The features with the largest discriminatory power between tissue types was Channel 2 (470 nm) lifetimes and Laguerre Coefficient with an effect of -0.83 and 0.98 respectively. Further, Channel 1 (390 nm) and PpIX (629 nm) intensity ratios had large effect sizes of -0.88 and 1.0 respectively (Fig. 6).

The multivariable model was developed similar to the in vivo data, starting with multi-leveled weighted principal component analysis (PCA) to reduce data dimension and multicollinearity (Supplemental Table 5). In the final multivariable model, features from both the autofluorescence and PpIX channels retained significance (*p* < 0.001). Lower lifetimes in Channel 2 (470 nm), lower intensity ratios in Channel 1 (390 nm) and higher intensity ratios in the PpIX Channel (629 nm) had the strongest association between FLIm features (predictor) and cancer (Table 3).

**Table 3:** Summary of coefficient estimates from weighted logistic mixed-effects model using the ex vivo data for predicting tissue type with FLIM parameters and principal components (PCs). Coefficient > 0 means that the higher value of the predictor, the higher probability that it is cancer; coefficient < 0 means that the higher value of the predictor, the lower probability that it is cancer; the magnitude of coefficient indicates the association between the predictor and tissue type (the larger, the stronger).

Predictor	Channel	Principal component	Coefficient (SE)	*P*-value
Intensity ratio	1 (390 nm)	-	-0.734 (0.023)	< 0.001
	2 (470 nm)	-	0.393 (0.042)	< 0.001
	3 (629 nm)	-	1.783 (0.043)	< 0.001
Lifetime average	1 (390 nm)	-	-0.154 (0.015)	< 0.001
	2 (470 nm)	-	-1.675 (0.038)	< 0.001
Phasor (Between-Subject PC)	2 (470 nm)	PC1	-0.791 (0.240)	< 0.001
Phasor (Within-Subject PC)	1 (390 nm)	PC1	0.004 (0.001)	< 0.001
		PC2	0.004 (0.001)	< 0.001
	3 (629 nm)	PC1	-0.532 (0.008)	< 0.001
		PC2	-0.302 (0.010)	< 0.001
Laguerre coefficient (Between-Subject PC)	3 (629 nm)	PC2	1.358 (0.353)	< 0.001
Laguerre coefficient (Within-Subject PC)	1 (390 nm)	PC1	-0.034 (0.004)	< 0.001
		PC2	-0.206 (0.007)	< 0.001
	2 (470 nm)	PC1	-0.149 (0.007)	< 0.001
		PC2	-0.093 (0.004)	< 0.001
	3 (629 nm)	PC1	-0.323 (0.006)	< 0.001
		PC2	0.635 (0.009)	< 0.001

### Machine learning based classifier output

**Fig. 7:**
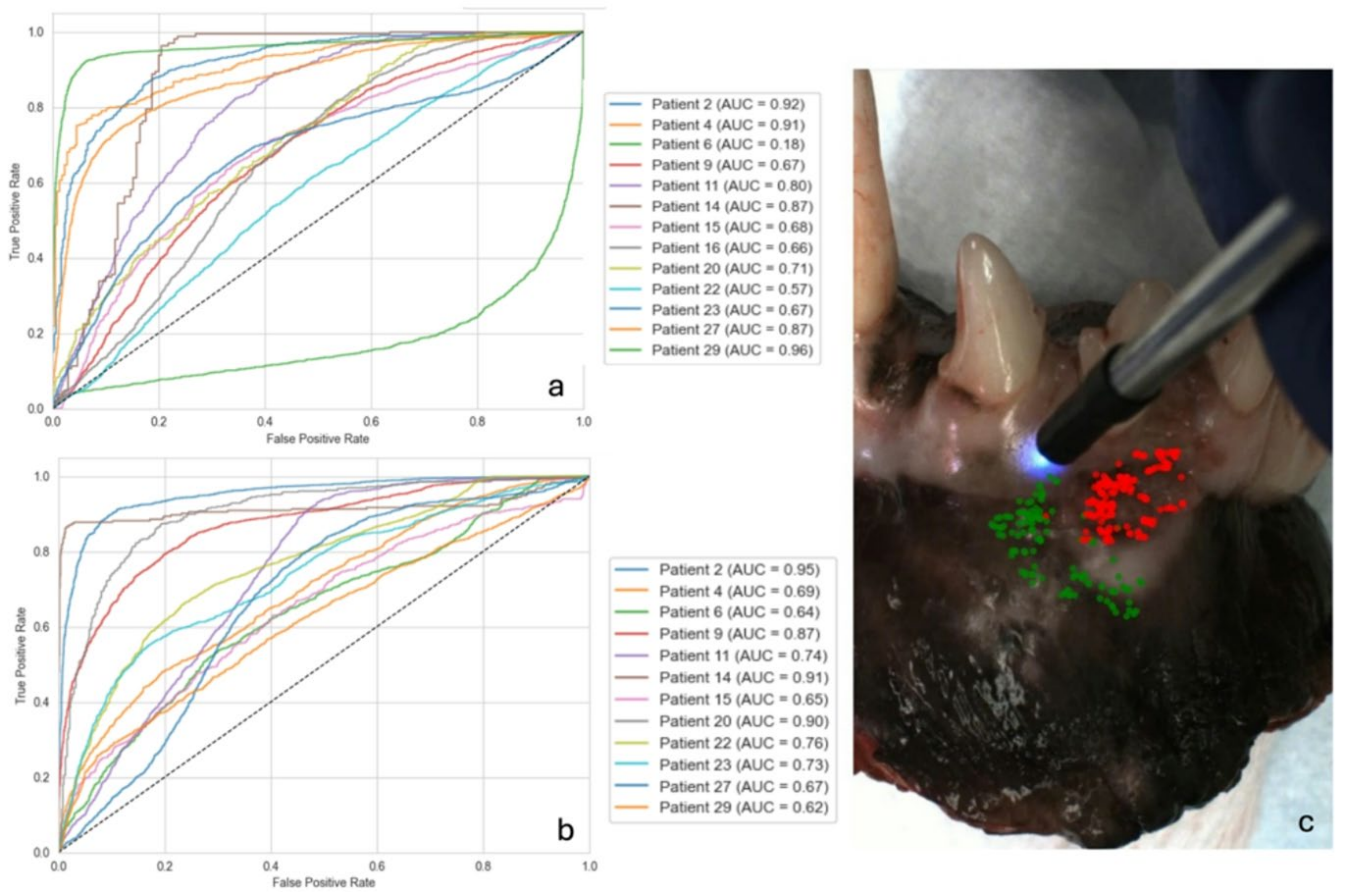
ROC analysis of a machine learning classifier using a multiparameter approach (combining multiple spectral features). ROC curve and associated AUC are shown for each patient in vivo (**a**) and ex vivo (**b**) scan. An example of the classifier applied to a patient sample (c). Note that there are a few false positives in the normal region; but the majority of data points were properly classified with the machine learning classifier (prediction of data points as cancer or healthy).

To further explore the ability for spectral features to differentiate between cancer and healthy data points within a single patient, we performed linear discrimination analysis. Patients 17 and 18 were scar revisions with no residual cancer, thus were excluded as interpatient tissue comparisons could not be performed. On the remaining patients linear discrimination analysis was performed using various combinations of spectral features for a model grid search. Based on the best performing AUC across all patients, the optimal model for both in and ex vivo tissue was a multiparameter evaluation of lifetimes + intensity ratios + laguerre coefficients + Phasors (Fig. 7).

For in vivo imaging, this model had a mean (S.D.) AUC across all patients of of 0.73 (0.20), accuracy of 0.71 (0.14), specificity of 0.71 (0.19), and sensitivity of 0.72 (0.23). Excluding patient 6 as an outlier the mean (S.D.) AUC was 0.77 (0.12). For ex vivo, this model had a mean (S.D.) AUC across all patients of 0.78 (0.13), accuracy of 0.74 (0.15), specificity of 0.78 (0.16), and sensitivity of 0.72 (0.21). No tumor type, tumor size, or broad anatomical location (maxilla vs. mandible, rostral vs. caudal) showed consistently better performance, rather performance appeared patient driven, with patients with a wide range of lifetimes in health performing worse. We propose that the range of lifetimes in healthy tissue are driven by spanning numerous tissue types in a single scan (i.e., from gingiva to mucosa or from inflamed to non-inflamed tissue).

As there was substantial overlap in Channel 3 lifetimes between healthy and normal data points (Fig. 5) suggesting potential uptake of PpIX in inflammatory healthy tissue we evaluated model performance excluding data from Channel 3. Exclusion of this channel decreased performance of the model (mean AUC 0.67), likely due to the positive contribution of Channel 3 intensity ratios to overall model performance in certain patients.

## Discussion

This is the first study to describe the use of FLIm for canine epithelial oral tumors establishing its initial feasibility to aid in real-time cancer delineation. Although, overlap of spectral features can be seen between healthy and cancer tissues, we found distinct trends in mean lifetimes and intensity ratios on a patient level, and showed that when the data points were combined across patients spectral tissue properties could discriminate tissue types both intraoperatively (in vivo*)* and chairside (ex vivo)*.* For in vivo FLIm imaging, autofluorescent spectral features had the best ability to discriminate tissue types, especially mean lifetimes in Channel 1 (390 nm). The addition of 5-ALA induced PpIX did not substantially improve diagnostic ability and dropped from the logistic regression model. This was also confirmed with application of a machine learning algorithm, which was not substantially improved with incorporation of the PpIX channel.

This is the first study to evaluate not only PpIX FLIm, but also the visual utility of 5-ALA induced PpIX for photodynamic diagnosis (PDD) in canine oral tumors. Similar to other studies evaluating the clinical utility of 5-ALA, there was a high sensitivity but moderate specificity in the ability to identify neoplastic tissue based on visual fluorescence to the naked eye^12,26,32–34^. Based on PCR, the accumulation of PpIX and associated visual fluorescence in canine oral SCC was primarily driven by increased production of the precursors of PpIX (HMBS, CPOX) as well as by upregulation of exogeneous 5-ALA transporters and endogenous 5-ALA production. Upregulation of 5-ALA transporters has also been identified as the primary driver of PpIX accumulation in human pancreatic, bladder, lung, and gastric cancer, yet PEPT1 was shown to be more impactful than the role of PEPT2 and PAT1 ^35–38^ Further, increased CPOX has been shown to be the driving force for increased PpIX when cutaneous carcinoma are treated with 5-ALA in addition to methotrexate or vitamin D3.^39,40^ The upregulation of HMBS may be novel to dogs or to carcinoma. Previous work in dogs revealed that carcinoma cell lines had a strong negative correlation between the FECH expression and accumulated PpIX^12^. Differences in our findings are most likely due to differences between the properties of in vivo and in vitro response of carcinoma to 5-ALA, as well as method differences between the two studies and how the gene expression was quantified (compared to normal tissue in the present study versus relative mRNA expression within each cell line). To the authors knowledge, there is no mechanistic data on PpIX accumulation in human oral SCC for comparison.

Clinically, although there was visual PpIX fluorescence seen with the naked eye in the oral tumors, it was still not possible to use this tool autonomously for surgical evaluation as the tumor margin was rarely clearly outlined and there was non-specific accumulation in other non-neoplastic sites. Surprisingly, the use of combinatorial imaging of PpIX with FLIm did not improve the accuracy allowing clinical application of this fluorophore for surgical guidance. Rather, PpIX lifetimes and associated spectral features were unable to discriminate cancer and normal tissue in vivo and were excluded from the final multivariant model. This may be due to secondary accumulation of PpIX in the surrounding inflamed tissues or endogenous porphyrins that are shifting spectral properties in this channel. Further evaluation of the 629- channel in healthy tissue revealed distinct differences in lifetimes between gingiva and mucosa suggesting that different tissue types may have preferential accumulation of PpIX or other endogenous tissue-specific fluorophores are being captured in this spectral region. Historical label-free FLIm studies in human HNSCC have excluded the 629 nm channel from evaluation^29–31^ and comprehensive fluorescent studies have never been performed in the canine oral cavity, thus the role of endogenous porphyrins that may be also emitting in this channel are unknown. One study evaluating optical properties in an in vivo hamster model showed lifetimes of approximately 6 ns in early carcinoma lesions with increased lifetimes of > 7 ns in advanced carcinoma^41^. Yet, this group did not report lifetimes for healthy mucosa, and these lifetimes are notably shorter then some of the lifetimes seen in the healthy canine tissues, suggesting a stronger role of endogensous poprhyrins in the canine gingiva and mucosa. Interestingly, the lack of discrimination between tissue types in Channel 3 was not present in the *ex-vivo* data set, especially regarding intensity ratios. This suggests that 5-ALA-induced PpIX may have more of a role for chairside imaging as this fluorophore appears to stay active in cancer tissue but not healthy inflamed tissue after excision. Future studies that explore a larger cohort of healthy oral tissue scans with and without the addition of oral 5-ALA are required to determine if accumulation of PpIX in healthy tissue or endogenous porphyrins are the primary cause of low discriminatory power in vivo and better define if it has a role for chairside imaging.

Alternatively, PpIX FLIm may have not contributed to tissue discrimination due to the shorter excitation wavelength used in our measurements (355 nm). Although, 355 nm has been shown to excite PpIX in neurosurgery, ^28^ and result in expected lifetimes with this excitation, the ideal Soret band and what is traditionally used for excitation of PpIX, is 405 nm.^27^ Thus, we may have underestimated the contribution of PpIX in tissue discrimination with this shorter, less efficient, excitation. We chose this excitation to capture both PpIX and autoflourescent signals (collagen, NADPH) simultaneously and then evaluated the relative intensity of PpIX by focusing in the 630 nm channel as previously reported by our group for glioma^42^. Yet, this lower excitation may have biased the data to show a stronger contribution of autoflourescent properties to tissue discrimination. However, given that fluorescence lifetimes are an intrinsic property of the flourophore and largely independent of excitation wavelength, this is unlikely. The use of intensity ratios rather than fluorescent intensity (which is dependent on excitation) also helps minimize the magnitude of a shorter excitation wavelength on the quality of the data.

Simultaneous evaluation of both endogenous and exogenous fluorophores was selected for ease of use and time of scan. By using a sole excitation within the pulsed laser system, we were able to scan the tumor and surrounding tumor region with the OR lights on in under 5 min in every patient, with most scans lasting 1 min or less (supplemental video 1). This easily was incorporated into surgical workflow. Further, this system is able to be incorporated with the operatory and room lights on ^43,44^, a distinct benefit compared to the use of PpIX or other flourophores with open field visual fluorescence that require no ambient background lights to be effective. To the authors’ knowledge, this is the only in vivo FLIm system currently being integrated in the operating room work-flow a for the assessment of tumor margins during head and neck cancer resection. While a recent study reported the use of an wide field FLIm system for endogenous tissue discrimination among cancerous, dysplastic, and normal tissues during oral examination, demonstrating strong contrast between tissue types,^45^ that work did not address the practical implications of limited field-of-view for intraoperative deployment or translation into the operative setting. Another study evaluated the clinical utility of combining FLIM with an exogenous fluorophore, indocyanine green, to discriminate solid tumors from healthy tissue, reporting high accuracy in ex-vivo specimen-based experiments^46^. However, the integration of this approach into real-time intraoperative decision-making remains unclear, particularly with respect to in vivo implementation and workflow constraints in the operating room.

Tissue types were best discriminated by focusing on autofluorescence properties rather than PpIX. For in vivo, Channel 1 lifetimes and Channel 2 intensity ratios had the strongest effect size and maintained significance in a multivariant model with other related spectral features (phasors and Laguerre coefficients). In the *ex-vivo* data set, there was a shift in the best discriminatory features with Channel 1 intensity ratios and Channel 2 lifetimes being more impactful. It is expected that the most relevant tissue properties will shift after excision of the specimen when there is rapid loss of blood supply and changes in metabolic properties. This finding has also been documented in human HNSCC^29^. However, despite the discriminatory power of autofluorescence features in both data sets, it is important to note that similar to channel 3, albeit to a much lesser extent, there was a variability in tissue spectral properties in both normal and cancer leading to overlap of data points between cancer and health in some patients. The range of lifetimes in healthy tissue appear to be driven by differences in the scanned tissue types with alveolar gingiva, especially when inflamed, having lower lifetimes in channel 1 and 2 compared to palatal gingiva and buccal mucosa. In humans, use of anatomy-specific classifiers has resulted in improved AUC, highlighting the importance of patient specific properties on the use of label-free FLIm^47^. Analysis of the data did not show distinct trends by broad anatomical location (rostral versus caudal, maxilla versus mandible) nor specific tumor type (POSCC, COSCC, CAA). This was evident in also applying the classifier, where in certain patients there was excellent AUC and others performed poorly. Further characterization is needed in dogs to assess the synergistic role of more defined anatomy and associated tissue properties to determine if the optimal tissue discrimination model should vary with specific tissue type (e.g. gingiva compared to mucosa).

Limitations of this work include the relatively small sample size with heterogenous epithelial oral tumors that spanned through multiple oral tissue types. Future work with an expanded data set is required to further evaluate the role of anatomy-specific sites and tumor specific features on label-free FLIm to allow for further optimization of data processing in canine oral cancer. Further, our methodology of assigning point level labels from tissue region labels has the potential to introduce registration errors secondary to tissue deformation during sectioning, differences between the images surface and the histology plane and manual alignment uncertainty. Since the points near the tumor-healthy interface are particularly prone to mislabeling these were often excluded from analysis and not given a label. Thus, important data points may have been lost; further, we cannot exclude that some data points were incorrectly coded which may have biased the data. We attempted to minimize this by having one author (SG) present at all stages of the procedure; yet the possibility for error cannot be excluded. Despite this, within our data set, on both a patient level and with all data combined we were able to use specific FLIm features to discriminate tissue types. Further, we were able to apply a machine learning algorithm which had good accuracy (AUC 0.73). This represtents the first study to evaluate the diagnostic accuracy of simultaneous use of label-free and PpIX FLIm for oral tumors in a spontaneous model of disease providing distinct insight that that label-free FLIm alone is sufficient to distinguish epithelial oral cancers from healthy tissue in dogs, and that the addition of exogenous markers such as 5-ALA–induced PpIX, does not substantially improve diagnostic accuracy. Thus, incorporation into further optimization work on the use of FLIm for head and neck cancer is not warranted.

## Methods

### PpIX fluorescence in canine oral cancer cell lines

Five canine oral cancer cell lines, including three canine acanthomatous ameloblastoma (CAA) and two squamous cell carcinoma (SCC) lines, were evaluated for 5-ALA-mediated PpIX fluorescence. All cell lines were established from tissue samples obtained from clinical patients at the Veterinary Medicine Teaching Hospital (UC Davis School of Veterinary Medicine, Davis, CA, IACUC #24338, owner consent on VMTH intake form) and were validated to be of canine origin and mycoplasma-free through STR based DNA profiling and multiplex PCR (IDEX Bioanalytics Cell Check Canine PLUS). Each cell line was grown in duplicate past passage 8 to serve as a positive (+ 5-ALA) and negative (− 5-ALA) control and evaluate the presence of autofluorescence in each cell line (*N* = 10).

PpIX accumulation was evaluated in both control and treated cell lines with flow cytometry and confocal microscopy. 5-ALA hydrochloride (Thermo Scientific, Switzerland) was dissolved in phosphate buffered saline and 10% fetal bovine serum and added to cell culture medium at a concentration of 5 mmol/L for 2 h prior to washing and evaluation of PpIX fluorescence as previously described^31^. FACScalibur (BD Biosciences) was utilized to assessing fluorescence on FL3 channel (488 nm excitation, 670 nm long-pass emission). For each independent experimental run, approximately 200,000 events were acquired and percent of fluorescent cells in the gate defined as positive was recorded. Flow cytometric data was analyzed with FlowJo v10.8.1 software (BD Biosciences).

For confocal fluorescence microscopy (Leica TCS SP8 STED 3X, Leica Microsystems, Wetzlar, Germany), 2 × 10^5^ cells were seeded on 14 mm microwell 1.5 cover glass bottom 35 mm dishes (Mattek, Ashland, MA) for 2 days prior to 5-ALA incubation. Following incubation, the cells were washed and suspended in FluoroBrite™ DMEM (Thermo Fisher Scientific) for imaging. Cells were excited at 470 nm for PpIX-fluorescence emission imaging at 670 nm. Confocal fluorescence imaging was conducted via 20x objective lens (NA = 0.75) with oil immersion. Throughout the experiment, the photomultiplier voltage, acquisition settings, and excitation light intensity remained constant. Integrated fluorescence intensity images were analyzed using NIH ImageJ software as previously described^32^. A total of 5 fields of view were imaged, from which the average integrated fluorescence intensity per cell line was derived. Statistics for cell line imaging and flow cytometry were computed with Prism software (GraphPad). Exact two-sided p-values were computed between positive and negative controls, with *p* < 0.05 considered significant.

### Animals

Fifteen pet dogs with oral tumors histopathologically confirmed as SCC or CAA were prospectively enrolled over a 15-month period. Inclusion criteria included pathologic confirmation of tumor type as SCC or CAA, consent to surgical intervention, and greater than 1 year of age. Exclusion criteria included other types of oral tumors, severe liver disease (liver enzymes > 2X elevated), severe kidney disease (Iris stage 3 or > ), anemia (HCT < 20%), neutropenia (< 2,000 neutrophils/ul), or any other clinically significant hematologic/biochemical abnormality grade 2 or higher (VCOG-CTAE v2). ^48^

All methods were carried out in accordance with relevant guidelines and regulations and reported in accordance with ARRIVE guidelines. The protocol was approved by the University of California-Davis Institutional Animal Care and Use Committee (IACUC #23110) and Institutional Clinical Trial Review Board (ICTRB). Signed client informed consent was obtained prior to enrollment. Animals were housed in the hospital during the study period (one night prior to surgery, 24–48 h post operatively), which is standard of care for oncologic surgery at our institution.

Three to four hours prior to FLIm imaging, dogs were administered 40 mg/kg 5-ALA orally, as previously reported^12^. The 5-ALA was stored, dosed, and suspended into sterile water for administration by the University of California Veterinary Pharmacy Department. Respiratory rate, heart rate, temperature, and clinical signs for adverse reaction were monitored after administration. All patient received maropitant (2 mg/kg PO) the night before administration per our anesthesia protocols. Following surgical removal of the oral tumors and post-operative monitoring patients were discharged and housed with their owners. A recheck examination was performed 10–14 days post-operatively and liver enzymes were assessed at this time to evaluate for any systemic side effects from the 5-ALA administration.

### Quantitative polymerase chain reaction (qPCR)

To describe the mechanistic reason for 5-ALA accumulation in canine oral SCC, qualitative PCR was performed. Two representative paraffin-embedded blocks were selected per patient: one that showed visual fluorescence of cancer tissue and a control (no fluorescence, no tumor tissue). For each target gene, (Supplemental Table 1), two primers and an internal hydrolysis fluorescent-labeled probe (5´ end, reporter dye FAM (6-carboxyfluorescein), 3´ end, quencher MGB (Minor Grove Binder)) were designed by our group using Primer Express software (Thermo Fisher Scientific, Waltham, MA). A Basic Local Alignment Search Tool (BLAST) of the amplicon confirmed unique species detection. Final quantitation was performed using the comparative Cq method (User Bulletin #2, Applied Biosystems) and reported as relative transcription or the n-fold difference relative to normal non-fluorescent tissue.

### Fluorescence imaging system

A custom-built fiber-based point scanning FLIm system (National Center for Interventional Biphotonic Technologies, Davis CA) was utilized for fluorescence lifetime imaging. Tissue was excited with a 355 nm pulsed laser (< 60 ps FWHM, 115 Hz repetition rate; micro-Q0 switched laser, STV-02E-140, TEEM Photonics, France) delivered through a 365-micrometer core multimode optical fiber (0.22 NA, FG365UEC, Thorlabs, USA). The 355 nm excitation wavelength was selected because it efficiently excites key autofluorescence markers associated with cancer metabolism (collagen, NAD(H), FAD) while also exciting PpIX. The fiber proximal end was coupled to a wavelength selection module (WSM) featuring 3 dichroic mirrors and bandpass filters to spectrally resolve the fluorescence signal into three distinct channels: (1) 390 +/- 20 nm which focused primarily on collagen autofluorescence which is often decreased in cancer due to extracellular matrix degradation, (2) 470 +/- 14 nm which focused primarily on NAD(P)H autofluorescence which is often decreased due to metabolic shifts and (3) 629 +/- 26.5 nm focused primarily on PpIX fluorescence which is expected to increase with PpIX accumulation in cancer. These wavelengths were selected based on previous work in human HNSCC and the known emission of PpIX^26^.

The spectrally separated fluorescence signals were detected using a pulse-sampling FLIm approach. The signals were directed to three variable-gain UV-enhanced silicon avalanche photodiode (Si APD) modules with integrated transimpedance amplifiers, ensuring high sensitivity across the selected spectral bands. ^44^ For real-time visualization of the measurement location, a 445 nm laser (TECBL-50G-440-USB-TTL, Worldstartech, Canada) was incorporated as an aiming beam, allowing precise guidance of fluorescence point measurements during post-processing. The fluorescence decay profiles were recorded using two two-channel digitizers (NI PXIe-5162, National Instruments, Austin, Texas), each with a 1.5 GHz bandwidth and a 2.5 GS/s per channel sampling rate, which provided the necessary temporal resolution to capture both short- and long-lifetime fluorescence signals.

### Imaging and pathology workflow

All tumors were imaged in the operating room (OR) under general anesthesia. Patients were all anesthetized and monitored by members of the anesthesiology department at the University of California Davis Veterinary Medical Teaching Hospital. All patients were induced with injectable anesthetics (propofol or alfaxalone intravenously) given to effect and maintained by inhaled anesthetics (isoflurane of sevoflurane) throughout the procedure. All patients received local regional blocks (infraorbital or maxillary or inferior alveolar pending location) prior to surgery with 0.5% bupivacaine. Additional analgesics were also administered throughout the procedure at the discretion of the anesthetist. No standard anesthetic plan was utilized across all animals due to the specific comorbidities, temperament, and invasiveness of surgery which differed across the patients.

Prior to surgical intervention the OR lights were turned off and blue light (405 nm,1000 mW mounted LED, 1000 mA with a 25.0 mm longpass filter with a cut on wavelength 450 nm ThorLabs, CO, USA) was applied to the tumor to observe the presence of PpIX visual fluorescence through a mounted video screen with a table side video camera (Chameleon3 USB3, Python 1300, Teledyne FLIR, Oregon, USA). This was marked as a binary yes/no for each tumor by gross visualization. The tumor and peri-tumor region (approximately 10 mm in every direction) were then scanned with the FLIm probe by a board-certified oral and maxillofacial surgeon (SG). To determine spatial location during scanning, a 445 nm continuous-wave aiming beam was utilized on the optical path and delivered to the tissue through the same fiber-optic probe. Each collected data point corresponds to signal collected form a single excitation pulse. The average size of the tissue volume excited by each pulse was approximately 0.5 mm diameter with 250-300- micron depth of penetration. The point measurement rate was 115 Hz, but the exact sampling density depends on the motion of the fiber probe by the surgeon (supplemental video 1). The tissue is typically oversampled along the scanning path by the surgeon since Nyquist sampling is achieved for a scanning speed of approximately 30 mm/s (1/2 x measurement rate x lateral resolution = 28.75).

Following imaging, surgery was performed per standard of care with 10 mm margins from the extent of neoplasia as seen on computed tomographic scan. Immediately following removal of the tumor, ex vivo scanning of the specimen was performed on the back table by a laboratory assistant.

**Fig. 8:**
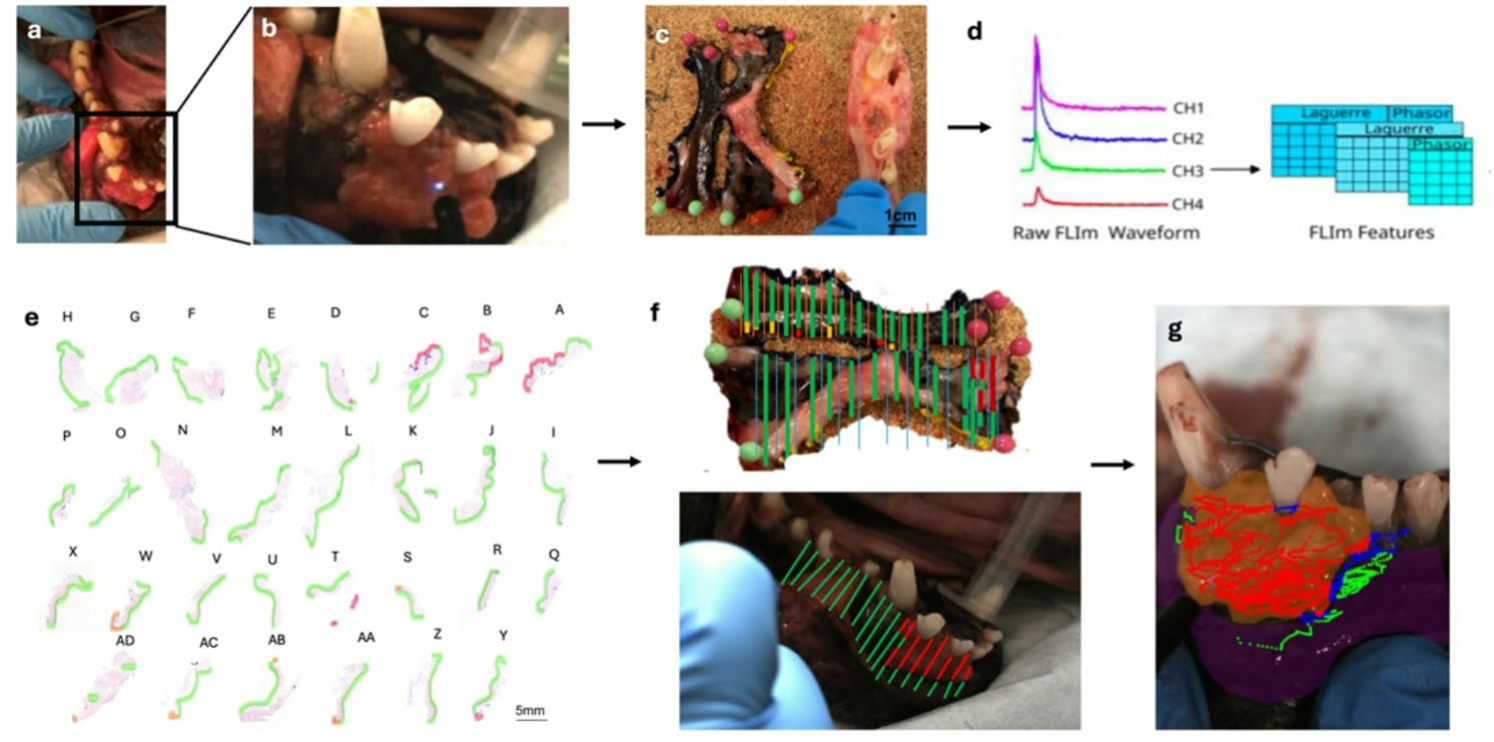
Overview of Fluorescence Lifetime Imaging (FLIm) workflow. (**a**) The tumor surface and surrounding normal tissue was scanned in vivo with a 355 nm pulsed laser to generate autofluorescence and excite PpIX. (**b**) Note the blue aiming beam, which allowed us to spatially register the data. (**c**) Following tumor excision, ex vivo scans were immediately performed and then the soft tissue was removed from the bone (if applicable). (**d**) Data was collected along three time-delayed spectral channels and deconvoluted to allow spectral parameters to be analyzed. (**e**) The tissue was serially sectioned every 5 mm, and the surface was annotated as normal (green), neoplasia (red), or dysplasia (yellow). (**f**) A pathology mask was created based on the annotations and co-registered with the video taken during FLIm acquisition. (**g**) This allowed all FLIm data points to be collated as cancer (red) or normal (green).

Prior to pathologic processing, the soft tissue was removed from the underlying osseous components of the mandible/maxilla (if applicable), pinned to a cork board mount to minimize deformation, and margins were inked. This allowed the soft tissue and resected jawbone to be processed separately, thereby avoiding loss of histological detail by the soft tissue during bone decalcification. After a minimum of 24 h in formalin, the soft tissue lesions were serially sectioned at 5 mm intervals and embedded in paraffin for histological slide preparation. Slides were digitized and the surface was annotated by a board-certified pathologist (NVA, BGM) as normal, dysplastic, or neoplastic tissue within 500 microns of the surface. Pathologists were blinded to the FLIm results. The surgeon (SG) was present for all pathology sectioning to confirm orientation and ensure accuracy. Each tissue section was measured and photographed to facilitate formation of a digital pathology map on a reference image of the ex-vivo specimen obtained prior to sectioning. The pathology map was then overlaid on still reference images from each in vivo and ex vivo FLIm video run utilizing anatomical features and tissue measurements to allow overlay of the pathology map on the still image. Regions where landmarks were ambiguous or where sectioning deformation prevented reliable alignment were excluded from analysis. We performed 2D spatial co-registration by manually assigning the pixel position of the FLIm measurement point on the corresponding white light video. The resulting region-level labels were transferred to FLIm point measurements falling within those mapped areas. Thus, point measurements that fell within each tissue region were defined as healthy or cancerous, respectively. The workflow is depicted in Fig. 8.

### Data processing

Data processing was performed in MATLAB (MathWorks, Inc. 2020, MATLAB R2020b Version 9.9. https://www.mathworks.com). The raw FLIm waveform was pre-processed with background subtraction to remove the fluorescence signal generated within the fiber probe and upsampled to 12.5 GS/s for enhanced temporal resolution.

Gain thresholding for each of the FLIm channels was determined using an iterative approach. For each channel, gain values were incremented in steps of 50 from 50 to 1000, and the corresponding mean fluorescence lifetime and standard deviation was evaluated. Thresholds were selected at the point where the standard deviation of the lifetime increased significantly, indicating a significant degradation in signal quality. This approach resulted in a gain threshold of > 15 and < 300 for channel 1, and < 800 for channel 2 and 3.

Two non-parametric techniques, Laguerre-based deconvolution^49^ and lifetime phasors^50^, were then utilized to derive the fluorescence decay characteristics for each spectral channel. Lifetimes were computed from the deconvolved fluorescence decay using the Laguerre expansion method^49^. Intensity ratios capture relative spectral contributions and were calculated for each FLIm channel by dividing the intensity of that channel by the sum of intensities of all channels. Phasor coordinates were derived from the Fourier transform of the decay to provide a model-free representation of lifetime behavior^50^. These metrics were selected because they are complementary, widely used FLIm descriptors that jointly capture spectral intensity, decay dynamics, and decay shape. Feature robustness was assessed by evaluating stability across repeated measurements and verifying consistency with standard fluorophore solution. The formulas utilized are presented in Supplementary Table 6. These methods generate multiple distinct spectral features per FLIm point, including 3 average lifetimes, 3 intensity ratios, 12 Laguerre coefficients, and 12 lifetime phasors.

The FLIm measurement locations were manually registered to the white light image resulting in x and y locations for a given scan. The scan points were excluded when the location of the aiming beam was not clear in the video. Although dysplasia was coded on the pathologic specimens, only binary annotations (cancer vs. non-cancer) were utilized for analysis.

### Statistical analysis

Separate analyses were performed for in vivo and ex vivo datasets for the 15 animals (including 13 with both cancer and healthy tissue types and 2 with only healthy type). Approximately 1% of in vivo observations and 4% of ex vivo observations were excluded from analysis due to missing values in FLIM parameters. For each dataset, all FLIM parameters were standardized (centered to zero-mean and scaled to unit standard deviation). For highly skewed FLIM parameters, square-root transformation was applied to improve normality before standardization. If there was any negative value in the FLIM parameter needing square-root transformation, a constant was added to all values of this FLIM parameter to ensure that the minimum value of this FLIM parameter is zero before the square-root transformation. To adjust for varying number of observations across animals and tissue types, we used weights in the following analysis. The weights were calculated based on inversed number of observations of each tissue type within each subject, and total summation of weights was the same as the sample size of dataset. Therefore, the weight-adjusted observations can map back to the original included dataset, and each animal represented equal sample size with half cancer and half healthy data points (except the 2 animals with only healthy tissue type, each of whom only represented half of data points as other animals).

We first performed univariable analysis to compare FLIM parameters between cancer and healthy tissue types. Weighted linear mixed-effects model was fitted for each FLIM parameter as outcome, including tissue type (cancer vs. healthy) as fixed effect, a random intercept to account for within-animal correlation, and weights to adjust for varying sample sizes across animals and tissue types. Effect sizes were calculated using Cohen’s d (=[Cancer mean - Healthy mean]/residual standard deviation), where the cancer vs. healthy mean differences and residual standard deviations were estimated from the fitted mixed-effects models. Effect sizes of d = ± 0.2 were considered small; d = ± 0.5 medium; and d = ± 0.8 large. P-values for estimated cancer vs. healthy mean differences were reported, along with adjusted p-values based on Benjamini-Hochberg procedure for multiple comparisons.

Due to many FLIM parameters and channels for Phasor and Laguerre coefficients, multi-leveled weighted principal component analysis (PCA) was performed for each channel of Phasor and Laguerre coefficient to reduce data dimension and multicollinearity. This method separated between-subject variation and within-subject variation by constructing between-subject principal components (PCs) (which captured heterogeneity across animals and did not vary within animals) and within-subject PCs (which captured variation within animals). For each Phasor and Laguerre parameter, weighted subject-level means were calculated for each animal, and then within-subject deviations (i.e., the difference between the parameter and its weighted subject-level mean) were calculated. The between-subject PCs were constructed by performing PCA using the weighted subject-level means. The within-subject PCs were constructed by performing PCA using the pooled within-subject deviations across animals. Two animals with only healthy tissue type were excluded during PCA, and their PCs were constructed using estimated PC loadings from PCA performed using the other 13 animals. First two principal components were used in the following analysis, which explained about 77% to 98% of variability. Univariable analysis was also performed to compare the within-subject PCs between cancer and healthy tissue types using weighted linear mixed-effects models.

Multivariable analysis was then conducted using weighted logistic mixed-effects models. The outcome was tissue type (cancer vs. healthy), and the model included a random intercept to account for within-animal correlation. Initial model included fixed effects of all intensity ratio, lifetime average parameters as well as between- and within-subjects PCs of Phasor and Laguerre coefficient. The model was further refined through a backward stepwise selection, removing one predictor at a time based on p-values and variance inflation factors (VIFs) to reduce collinearity and address statistical non-significance. Specifically, we prioritized removing those predictors that exhibited the most severe multicollinearity (VIF > 10). After resolving multicollinearity, we then sequentially removed remaining predictors with large p-values until all remaining predictors had p-values > 0.05. The estimated coefficients in the final fitted logistic model were reported. Coefficient > 0 means that the higher value of the predictor, the higher probability that it is cancer; coefficient < 0 means that the higher value of the predictor, the lower probability that it is cancer; the magnitude of coefficient indicates the association between the predictor and tissue type (the larger, the stronger). The coefficients for within-subject PCs indicate their ability to differentiate cancer vs. healthy tissues within the animal.

Computer based machine learning multiparameter discrimination was performed using a decision tree-based classification model as previously described^35^. Two- fold model optimization was performed^51^ hyper-parameter optimization using Bayesian optimization (Supplementary Table 7) and FLIm feature selection using a grid-search approach on multiple FLIm feature combinations. Classification was performed for each patient using the optimal classification model developed for each scan type (i.e., in vivo*/ *ex vivo). The findings were then summarized across all patients using mean, median, and standard deviation to define the optimal model for discrimination in canine epithelial oral cancer.

## Supplementary Information

Below is the link to the electronic supplementary material.

Supplementary Material 1

Supplementary Material 2

## Data Availability

The raw spectral dataset and accompanying processed, filtered, and annotated data generated and analyzed during the current study are available from the corresponding author on reasonable request.

## References

[1] Long, S. M. et al. Use of intraoperative frozen section to assess final tumor margin status in patients undergoing surgery for oral cavity squamous cell carcinoma. *JAMA Otolaryngol. Head Neck Surg.* **148**, 911–917 (2022). 35925571 10.1001/jamaoto.2022.2131 PMC9353701

[2] Horwich, P. et al. Specimen oriented intraoperative margin assessment in oral cavity and oropharyngeal squamous cell carcinoma. *J. Otolaryngol. Head Neck Surg.* **50**, 37 (2021). 34154663 10.1186/s40463-021-00501-5 PMC8218466

[3] Kerawala, C. J. & Ong, T. K. Relocating the site of frozen sections–is there room for improvement? *Head Neck*. **23**, 230–232 (2001). 11428454 10.1002/1097-0347(200103)23:3<230::aid-hed1023>3.0.co;2-v

[4] Miller, A. et al. How Far are we off? Analyzing the accuracy of surgical margin relocation in the head and neck. *Head Neck*. **46**, 2709–2716 (2024). 38702976 10.1002/hed.27793 PMC12032842

[5] Orosco, R. K. et al. Positive surgical margins in the 10 most common solid cancers. *Sci. Rep.* **8**, 5686 (2018). 29632347 10.1038/s41598-018-23403-5 PMC5890246

[6] Culp, W. T. N. et al. Results of surgical excision and evaluation of factors associated with survival time in dogs with lingual neoplasia: 97 cases (1995–2008). *J. Am. Vet. Med. Assoc.* **242**, 1392–1397 (2013). 23634684 10.2460/javma.242.10.1392

[7] Willenbrink, T. J. et al. Field cancerization: Definition, epidemiology, risk factors, and outcomes. *J. Am. Acad. Dermatol.* **83**, 709–717 (2020). 32387665 10.1016/j.jaad.2020.03.126

[8] Goldschmidt, S. Surgical margins for ameloblastoma in dogs: A review with an emphasis on the future. *Front. Vet. Sci.* **9**, 830258 (2022). 35392113 10.3389/fvets.2022.830258 PMC8980539

[9] Judy, R. P. et al. Quantification of tumor fluorescence during intraoperative optical cancer imaging. *Sci. Rep.* **5**, 16208 (2015). 26563091 10.1038/srep16208 PMC4643322

[10] Holt, D. et al. Intraoperative near-infrared imaging can distinguish cancer from normal tissue but not inflammation. *PLoS ONE*. **9**, e103342 (2014). 25072388 10.1371/journal.pone.0103342 PMC4114746

[11] Holt, D. et al. Intraoperative near-infrared fluorescence imaging and spectroscopy identifies residual tumor cells in wounds. *J. Biomed. Opt.* **20**, 76002 (2015). 26160347 10.1117/1.JBO.20.7.076002 PMC4497968

[12] Osaki, T. et al. Efficacy of 5-Aminolevulinic acid in photodynamic detection and photodynamic therapy in veterinary medicine. *Cancers (Basel)* **11**, (2019). 10.3390/cancers11040495 PMC6520946 30959982

[13] de Boer, E. et al. Optical innovations in surgery. *Br. J. Surg.* **102**, e56–72 (2015). 25627136 10.1002/bjs.9713

[14] Debie, P. & Hernot, S. Emerging fluorescent molecular tracers to guide Intra-Operative surgical Decision-Making. *Front. Pharmacol.* **10**, 510 (2019). 31139085 10.3389/fphar.2019.00510 PMC6527780

[15] Pan, J. et al. Real-time surveillance of surgical margins via ICG-based near-infrared fluorescence imaging in patients with OSCC. *World J. Surg. Oncol.* **18**, 96 (2020). 32414418 10.1186/s12957-020-01874-z PMC7229610

[16] Nishio, N. et al. Optimal dosing strategy for Fluorescence-Guided surgery with Panitumumab-IRDye800CW in head and neck cancer. *Mol. Imaging Biol.* **22**, 156–164 (2020). 31054001 10.1007/s11307-019-01358-x PMC7017887

[17] Morlandt, A. B. et al. Fluorescently labeled Cetuximab-IRDye800 for guided surgical excision of ameloblastoma: A proof of principle study. *J. Oral Maxillofac. Surg.* **78**, 1736–1747 (2020). 32554066 10.1016/j.joms.2020.05.022 PMC7541684

[18] Gao, R. W. et al. Determination of tumor margins with surgical specimen mapping using Near-Infrared fluorescence. *Cancer Res.* **78**, 5144–5154 (2018). 29967260 10.1158/0008-5472.CAN-18-0878 PMC6125224

[19] Fluorescence Lifetime Spectroscopy and Imaging. (CRC, 2014). 10.1201/b17018

[20] Lutz, V. et al. Impact of collagen crosslinking on the second harmonic generation signal and the fluorescence lifetime of collagen autofluorescence. *Skin. Res. Technol.* **18**, 168–179 (2012). 21564311 10.1111/j.1600-0846.2011.00549.x

[21] Blinova, K. et al. Distribution of mitochondrial NADH fluorescence lifetimes: steady-state kinetics of matrix NADH interactions. *Biochemistry* **44**, 2585–2594 (2005). 15709771 10.1021/bi0485124

[22] Lakowicz, J. R., Szmacinski, H., Nowaczyk, K. & Johnson, M. L. Fluorescence lifetime imaging of free and protein-bound NADH. *Proc. Natl. Acad. Sci. USA*. **89**, 1271–1275 (1992). 1741380 10.1073/pnas.89.4.1271 PMC48431

[23] Islam, M. S., Honma, M., Nakabayashi, T., Kinjo, M. & Ohta, N. pH dependence of the fluorescence lifetime of FAD in solution and in cells. *Int. J. Mol. Sci.* **14**, 1952–1963 (2013). 23334475 10.3390/ijms14011952 PMC3565358

[24] Kelty, C. J., Brown, N. J., Reed, M. W. R. & Ackroyd, R. The use of 5-aminolaevulinic acid as a photosensitiser in photodynamic therapy and photodiagnosis. *Photochem. Photobiol Sci.* **1**, 158–168 (2002). 12659511 10.1039/b201027p

[25] McNicholas, K., MacGregor, M. N. & Gleadle, J. M. In order for the light to shine so brightly, the darkness must be present-why do cancers fluoresce with 5-aminolaevulinic acid? *Br. J. Cancer*. **121**, 631–639 (2019). 31406300 10.1038/s41416-019-0516-4 PMC6889380

[26] Lima, I. F. P., Brand, L. M., de Figueiredo, J. A. P., Steier, L. & Lamers, M. L. Use of autofluorescence and fluorescent probes as a potential diagnostic tool for oral cancer: A systematic review. *Photodiagnosis Photodyn Ther.* **33**, 102073 (2021). 33232819 10.1016/j.pdpdt.2020.102073

[27] Reichert, D. et al. Fluorescence lifetime imaging and spectroscopic Co-Validation for protoporphyrin IX-Guided tumor visualization in neurosurgery. *Front. Oncol.* **11**, 741303 (2021). 34595120 10.3389/fonc.2021.741303 PMC8476921

[28] Alfonso-Garcia, A. et al. Mesoscopic fluorescence lifetime imaging: fundamental principles, clinical applications and future directions. *J. Biophotonics*. **14**, e202000472 (2021). 33710785 10.1002/jbio.202000472 PMC8579869

[29] Marsden, M. et al. Intraoperative margin assessment in oral and oropharyngeal cancer using Label-Free fluorescence lifetime imaging and machine learning. *IEEE Trans. Biomed. Eng.* **68**, 857–868 (2021). 32746066 10.1109/TBME.2020.3010480 PMC8960054

[30] Weyers, B. W. et al. Fluorescence lifetime imaging for intraoperative cancer delineation in transoral robotic surgery. *Translational Biophotonics* **1**, (2019). 10.1002/tbio.201900017 PMC7351319 32656529

[31] Hassan, M. A. et al. FLIm-Based in Vivo Classification of Residual Cancer in the Surgical Cavity During Transoral Robotic Surgery. In: *Medical image computing and computer assisted intervention – MICCAI 2023: 26th international conference, vancouver, BC, canada, october 8–12*, proceedings, part IX (eds. Greenspan, H.) 14228 587–596 (Springer Nature Switzerland, 2023). 10.1007/978-3-031-43996-4_56 PMC12349881 40810134

[32] Stepp, H. & Stummer, W. 5-ALA in the management of malignant glioma. *Lasers Surg. Med.* **50**, 399–419 (2018). 29737540 10.1002/lsm.22933

[33] Broekx, S., Weyns, F. & De Vleeschouwer 5-Aminolevulinic acid for recurrent malignant gliomas: A systematic review. *Clin. Neurol. Neurosurg.* **195**, 105913 (2020). 32447151 10.1016/j.clineuro.2020.105913

[34] Howley, R., Chandratre, S. & Chen, B. 5-Aminolevulinic acid as a theranostic agent for tumor fluorescence imaging and photodynamic therapy. *Bioengineering (Basel)* **10**, (2023). 10.3390/bioengineering10040496 PMC10136048 37106683

[35] Labib, P. L., Yaghini, E., Davidson, B. R., MacRobert, A. J. & Pereira, S. P. 5-Aminolevulinic acid for fluorescence-guided surgery in pancreatic cancer: cellular transport and fluorescence quantification studies. *Transl. Oncol.* **14**, 100886 (2020). 33059124 10.1016/j.tranon.2020.100886 PMC7566921

[36] Hagiya, Y. et al. Expression levels of PEPT1 and ABCG2 play key roles in 5-aminolevulinic acid (ALA)-induced tumor-specific protoporphyrin IX (PpIX) accumulation in bladder cancer. *Photodiagnosis Photodyn. Ther.* **10**, 288–295 (2013). 23993855 10.1016/j.pdpdt.2013.02.001

[37] Hagiya, Y. et al. Pivotal roles of peptide transporter PEPT1 and ATP-binding cassette (ABC) transporter ABCG2 in 5-aminolevulinic acid (ALA)-based photocytotoxicity of gastric cancer cells in vitro. *Photodiagnosis Photodyn. Ther.* **9**, 204–214 (2012). 22959800 10.1016/j.pdpdt.2011.12.004

[38] Omoto, K. et al. Expression of peptide transporter 1 has a positive correlation in protoporphyrin IX accumulation induced by 5-aminolevulinic acid with photodynamic detection of non-small cell lung cancer and metastatic brain tumor specimens originating from non-small cell lung cancer. *Photodiagnosis Photodyn. Ther.* **25**, 309–316 (2019). 30639584 10.1016/j.pdpdt.2019.01.009

[39] Anand, S., Wilson, C., Hasan, T. & Maytin, E. V. Vitamin D3 enhances the apoptotic response of epithelial tumors to aminolevulinate-based photodynamic therapy. *Cancer Res.* **71**, 6040–6050 (2011). 21807844 10.1158/0008-5472.CAN-11-0805 PMC3360482

[40] Anand, S., Honari, G., Hasan, T., Elson, P. & Maytin, E. V. Low-dose methotrexate enhances aminolevulinate-based photodynamic therapy in skin carcinoma cells in vitro and in vivo. *Clin. Cancer Res.* **15**, 3333–3343 (2009). 19447864 10.1158/1078-0432.CCR-08-3054 PMC2744072

[41] Sun, Y. et al. In vivo validation of a bimodal technique combining time-resolved fluorescence spectroscopy and ultrasonic backscatter microscopy for diagnosis of oral carcinoma. *J. Biomed. Opt.* **17**, 116003 (2012). 23117798 10.1117/1.JBO.17.11.116003 PMC3484195

[42] Alfonso-García, A. et al. First in patient assessment of brain tumor infiltrative margins using simultaneous time-resolved measurements of 5-ALA-induced PpIX fluorescence and tissue autofluorescence. *J. Biomed. Opt.* **27**, (2022). 10.1117/1.JBO.27.2.020501 PMC8809358 35112514

[43] Zhou, X., Bec, J., Ehrlich, K., Garcia, A. A. & Marcu, L. Pulse-sampling fluorescence lifetime imaging: evaluation of photon economy. *Opt. Lett.* **48**, 4578–4581 (2023). 37656559 10.1364/OL.490096 PMC10883700

[44] Zhou, X., Bec, J., Yankelevich, D. & Marcu, L. Multispectral fluorescence lifetime imaging device with a silicon avalanche photodetector. *Opt. Express*. **29**, 20105–20120 (2021). 34266107 10.1364/OE.425632 PMC8237936

[45] Duran-Sierra, E. et al. Clinical label-free endoscopic imaging of biochemical and metabolic autofluorescence biomarkers of benign, precancerous, and cancerous oral lesions. *Biomed. Opt. Express*. **13**, 3685–3698 (2022). 35991912 10.1364/BOE.460081 PMC9352301

[46] Pal, R. et al. Fluorescence lifetime of injected indocyanine green as a universal marker of solid tumours in patients. *Nat. Biomed. Eng.* **7**, 1649–1666 (2023). 37845517 10.1038/s41551-023-01105-2

[47] Hassan, M. A. et al. Anatomy-Specific classification model using Label-Free flim to aid intraoperative surgical guidance of head and neck cancer. *IEEE Trans. Biomed. Eng.* **70**, 2863–2873 (2023). 37043314 10.1109/TBME.2023.3266678 PMC10833893

[48] LeBlanc, A. K. et al. Veterinary cooperative oncology Group-Common terminology criteria for adverse events (VCOG-CTCAE v2) following investigational therapy in dogs and cats. *Vet. Comp. Oncol.* **19**, 311–352 (2021). 33427378 10.1111/vco.12677 PMC8248125

[49] Liu, J., Sun, Y., Qi, J. & Marcu, L. A novel method for fast and robust estimation of fluorescence decay dynamics using constrained least-squares Deconvolution with Laguerre expansion. *Phys. Med. Biol.* **57**, 843–865 (2012). 22290334 10.1088/0031-9155/57/4/843 PMC3407553

[50] Fereidouni, F., Gorpas, D., Ma, D., Fatakdawala, H. & Marcu, L. Rapid fluorescence lifetime Estimation with modified phasor approach and Laguerre deconvolution: a comparative study. *Methods Appl. Fluoresc*. **5**, 035003 (2017). 28644150 10.1088/2050-6120/aa7b62 PMC6043162

[51] Hassan, M. A. et al. Enhancing label-free fluorescence lifetime imaging for intraoperative tumor margin delineation in head and neck cancer using Data-Centric AI. *Res. Sq*. 10.21203/rs.3.rs-6673642/v1 (2025).41472684

