# Supplementary material for: Label-free fluorescence lifetime imaging can distinguish cancer from healthy tissue in spontaneously occurring canine oral tumors
Source: Sci Rep. 2026 Jan 23;16:6077. doi: 10.1038/s41598-026-37001-3 (PMC12902045; doi:10.1038/s41598-026-37001-3)
Supplement: Supplementary file 2 — Supplementary Material 2 [file 41598_2026_37001_MOESM2_ESM.pdf]

Label-Free Fluorescence Lifetime Imaging (FLIm) is Superior to 5-ALA-Induced Protophoryin IX FLIm to Distinguish Cancer From Healthy Tissue in Spontaneously Occurring Canine Oral Tumors

Stephanie Goldschmidt[1*], Laura Marcu[2], Katjana Ehrlich[2], Mohamed Abul Hassan[2], Iris Rivas[1], Andrew Birkeland[3] Xiangnan Zhou[2], Julien Bec[2], Alba Alfonso Garcia[2], Shuai Chen[4], Yichu Chen[5], Yash Tipirneni[1], Max Kampe[1], Abigail Weir[1], Abraham Morales[1], Chrisine Ly[6] ,Robert Rebhun[1] Brian G. Murphy[6], Natalia Vapniarsky[6]

Supplementary Media

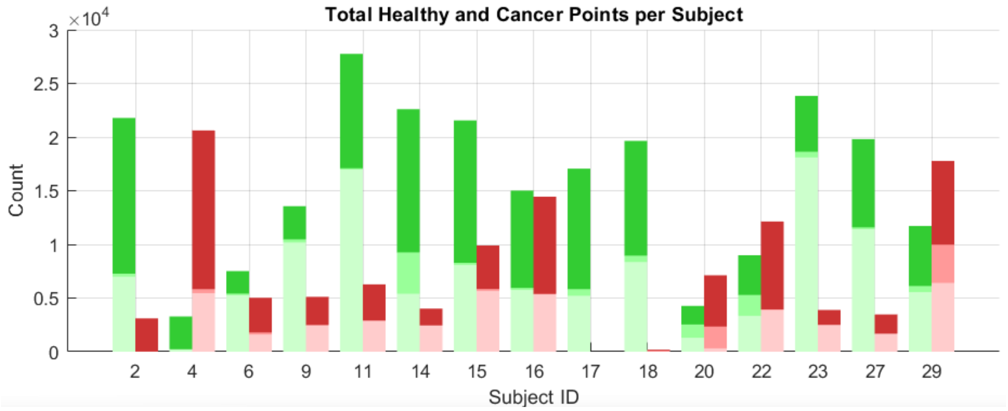

Supplementary Figure 1. Total number of healthy cancer and data points obtained per patient. The total bar represents all healthy (green) and cancer (red) data points collected per patient. The brighter red/green are data points that were excluded after data processing (gain and SNR thresholding and ensuring label was present for each point).

Supplementary Video 1. Video example of an intraoperative scan of a tumor in a clinical patient to demonstrate the speed of data acquisition. Traditionally the gross tumor and peri-tumoral region were acquired in different scans for ease of data naming.

Supplementary Tables

Supplementary Table 1: **Mechanistic action of 5-ALA induced PpIX accumulation in canine oral squamous cell carcinoma (SCC) quantified through RT-PCR analysis.** Differing gene expression in eight patients between high flourescence SCC samples and non-flourescent normal tissue. Note patient 4 had viral papillomas and SCC (presumed malignant transformation but no termporal proof of this relationship); 4a represents a flourescent papilloma and 4b represents flourescent neoplastic tissue. Below the RT-PCR the primer probes utilized for each gene evaluated in the heme biosynthetic pathway are shown.

Patient	ALAS1	ALAD	HMBS	UROS	CPOX	PPOX	FECH	SLC15A1	SLC15A2	SLC6A13
9	1.50	-1.09	2.19	-1.08	3.23	1.10	1.73	8.49	2.08	3.75
6	1.25	-2.62	1.17	-4.66	2.22	-3.65	-2.56	Not expressed	2.64	5.43
11	3.09	-1.42	1.35	-1.43	1.09	1.33	1.21	2.24	1.44	1.99
4a	-4.85	-17.47	-5.20	-8.56	-2.02	-4.75	-7.25	Expressed	-19.24	-3.48
4b	-2.32	-5.96	-2.52	-4.52	1.75	-1.99	-2.52	Expressed	-2.55	2.40
14	2.02	2.31	1.50	1.63	4.28	1.48	1.52	-1.79	5.97	1.18
15	-1.81	-1.56	1.32	-3.49	1.83	-3.38	-1.32	Expressed	1.36	3.41
20	1.48	3.45	6.97	6.97	3.80	2.91	5.82	Not expressed	3.44	-1.86
23	4.75	6.52	5.93	2.71	4.47	3.86	3.07	Not expressed	8.60	17.28

Gene	Assay order number	NCBI gene sequence
ALAS 1	Cf02731373_m1	XM_014122254.2
ALAD	Cf02692751_m1	XM_850277.5
HMBS	Cf02694649_g1	XM_005619681.3
UROS	Cf02661523_g1	XM_005637838.3
CPOX	Cf02692825_m1	XM_005639374.3
PPOX	Cf02690625_gH	XM_005640873.2
FECH	Cf02692851_m1	XM_022416725.1
PEPT 1 (SLC15A1)	Cf02703983_m1	NM_001003036.1
PEPT 2 (SLC15A2)	Cf02741721_m1	XM_022413842.1
HPRT1	Cf02690456_g1	NM_001003357.2

Supplemental Table 2. Univariate comparison of FLIm parameters between cancer and healthy tissues using the *in vivo* data set. Summary data is based on univariable analysis with a weighted linear mixed-effects models for each FLIm parameter with tissue type (healthy versus cancer) as the fixed effect and a random intercept to account for within-animal correlation. [c] Square-root transformation was applied to improve normality of the annotated FLIm parameter before standardization

FLIM Parameter	Channel	Effect Size (Cohen's d)	P-value	Adjusted P-value
Intensity Ratio	1[c]	-0.51	<0.001	<0.001
	2	-0.65	<0.001	<0.001
	3	0.61	<0.001	<0.001
Lifetime Average	1	-0.74	<0.001	<0.001
	2	-0.45	<0.001	<0.001
	3	0.13	<0.001	<0.001
Phasor (real component)	1-1[c]	0.01	0.092	0.092
	1-2[c]	0.15	<0.001	<0.001
	1-3[c]	0.27	<0.001	<0.001
	1-4[c]	0.35	<0.001	<0.001
	2-1	-0.06	<0.001	<0.001
	2-2	0.15	<0.001	<0.001
	2-3	0.35	<0.001	<0.001
	2-4	0.46	<0.001	<0.001
	3-1	-0.07	<0.001	<0.001
	3-2	-0.15	<0.001	<0.001
	3-3	-0.16	<0.001	<0.001
	3-4	-0.15	<0.001	<0.001
Phasor (imag component)	1-1[c]	-0.59	<0.001	<0.001
	1-2[c]	-0.65	<0.001	<0.001
	1-3[c]	-0.62	<0.001	<0.001
	1-4[c]	-0.58	<0.001	<0.001
	2-1	-0.37	<0.001	<0.001
	2-2	-0.60	<0.001	<0.001
	2-3	-0.74	<0.001	<0.001
	2-4	-0.80	<0.001	<0.001
	3-1[c]	0.20	<0.001	<0.001
	3-2[c]	0.16	<0.001	<0.001
	3-3[c]	0.07	<0.001	<0.001
	3-4[c]	-0.20	<0.001	<0.001
Laguerre Coefficient	1-1	0.02	0.002	0.002
	1-2	0.55	<0.001	<0.001
	1-3	0.05	<0.001	<0.001
	1-4	0.41	<0.001	<0.001
	1-5	-0.04	<0.001	<0.001
	1-6	0.21	<0.001	<0.001
	1-7	-0.23	<0.001	<0.001
	1-8	0.10	<0.001	<0.001
	1-9	-0.29	<0.001	<0.001
	1-10	-0.08	<0.001	<0.001
	1-11	-0.36	<0.001	<0.001
	1-12	-0.20	<0.001	<0.001
	2-1	-0.05	<0.001	<0.001

	2-2	0.55	<0.001	<0.001
	2-3	-0.37	<0.001	<0.001
	2-4	0.52	<0.001	<0.001
	2-5	-0.17	<0.001	<0.001
	2-6	0.32	<0.001	<0.001
	2-7	-0.08	<0.001	<0.001
	2-8	0.20	<0.001	<0.001
	2-9	-0.04	<0.001	<0.001
	2-10	0.20	<0.001	<0.001
	2-11	-0.16	<0.001	<0.001
	2-12	0.27	<0.001	<0.001
	3-1	0.03	<0.001	<0.001
	3-2	-0.08	<0.001	<0.001
	3-3	0.17	<0.001	<0.001
	3-4	-0.10	<0.001	<0.001
	3-5	0.09	<0.001	<0.001
	3-6	-0.08	<0.001	<0.001
	3-7	0.02	0.008	0.008
	3-8	-0.08	<0.001	<0.001
	3-9	-0.03	<0.001	<0.001
	3-10[c]	-0.07	<0.001	<0.001
	3-11	0.06	<0.001	<0.001
	3-12	0.13	<0.001	<0.001

Supplemental Table 3. Comparisons of within-subject principal components (PCs) of phasor and Laguerre coefficient parameters using the *in vivo* data based on univariable analysis with weighted linear mixed-effects models. Multi-leveled principal component analysis was used to construct principle components (PCs) for each Phasor or Laguerre Coefficient channel, with between-subject PCs (which reflect heterogeneity across animals) and within-subject PCs (which reflect variation within animals) to identify which spectral features to include in the multivariable model.

Within-subject principal component	Channel	Principal Component	Effect Size (Cohen's d)	P-value	Adjusted P-value
Phasor	1	PC1	-0.71	<0.001	<0.001
		PC2	-0.21	<0.001	<0.001
	2	PC1	-0.36	<0.001	<0.001
		PC2	-0.65	<0.001	<0.001
	3	PC1	-0.21	<0.001	<0.001
		PC2	0.10	<0.001	<0.001
Laguerre Coefficient	1	PC1	-0.11	<0.001	<0.001
		PC2	0.64	<0.001	<0.001
	2	PC1	0.18	<0.001	<0.001
		PC2	0.09	<0.001	<0.001

	3	PC1	0.11	<0.001	<0.001
		PC2	-0.13	<0.001	<0.001

Supplemental Table 4. Univariate comparison of FLIm parameters between cancer and healthy tissues using the *ex vivo* data set. Summary data is based on univariable analysis with a weighted linear mixed-effects models for each FLIm parameter with tissue type (healthy versus cancer) as the fixed effect and a random intercept to account for within-animal correlation. [c] Square-root transformation was applied to improve normality of the annotated FLIm parameter before standardization

**FLIM Parameter**	**Channel**	**Effect Size (Cohen's d)**	**P-value**	**Adjusted P-value**
Intensity Ratio	1[c]	-0.88	<0.001	<0.001
	2	-0.43	<0.001	<0.001
	3[c]	1.00	<0.001	<0.001
Lifetime Average	1	-0.23	<0.001	<0.001
	2	-0.83	<0.001	<0.001
	3	0.53	<0.001	<0.001
Phasor (real component)	1-1[c]	0.00	0.834	0.847
	1-2[c]	0.00	0.886	0.886
	1-3[c]	0.00	0.736	0.771
	1-4[c]	0.00	0.609	0.648
	2-1	0.03	<0.001	<0.001
	2-2	0.44	<0.001	<0.001
	2-3	0.69	<0.001	<0.001
	2-4	0.83	<0.001	<0.001
	3-1	-0.48	<0.001	<0.001
	3-2	-0.55	<0.001	<0.001
	3-3	-0.50	<0.001	<0.001
	3-4	-0.44	<0.001	<0.001
Phasor (imag component)	1-1[c]	-0.11	<0.001	<0.001
	1-2[c]	-0.12	<0.001	<0.001
	1-3[c]	-0.11	<0.001	<0.001
	1-4[c]	-0.13	<0.001	<0.001
	2-1	-0.70	<0.001	<0.001
	2-2	-1.00	<0.001	<0.001
	2-3	-1.16	<0.001	<0.001
	2-4	-1.22	<0.001	<0.001
	3-1	0.34	<0.001	<0.001
	3-2	0.28	<0.001	<0.001
	3-3[c]	-0.18	<0.001	<0.001
	3-4[c]	-0.80	<0.001	<0.001
Laguerre Coefficient	1-1	-0.69	<0.001	<0.001
	1-2[c]	-0.05	<0.001	<0.001
	1-3[c]	-0.63	<0.001	<0.001
	1-4	0.00	0.749	0.772
	1-5[c]	-0.55	<0.001	<0.001

	1-6	-0.01	0.243	0.263
	1-7[c]	-0.52	<0.001	<0.001
	1-8	-0.03	<0.001	<0.001
	1-9[c]	-0.51	<0.001	<0.001
	1-10	-0.08	<0.001	<0.001
	1-11[c]	-0.53	<0.001	<0.001
	1-12	-0.06	<0.001	<0.001
	2-1	-0.06	<0.001	<0.001
	2-2	0.98	<0.001	<0.001
	2-3	-0.59	<0.001	<0.001
	2-4	0.79	<0.001	<0.001
	2-5	-0.58	<0.001	<0.001
	2-6	0.47	<0.001	<0.001
	2-7	-0.55	<0.001	<0.001
	2-8	0.32	<0.001	<0.001
	2-9	-0.52	<0.001	<0.001
	2-10	0.33	<0.001	<0.001
	2-11	-0.56	<0.001	<0.001
	2-12	0.48	<0.001	<0.001
	3-1	0.02	0.002	0.002
	3-2	-0.08	<0.001	<0.001
	3-3	0.58	<0.001	<0.001
	3-4	-0.05	<0.001	<0.001
	3-5	0.60	<0.001	<0.001
	3-6	0.02	0.012	0.013
	3-7	0.47	<0.001	<0.001
	3-8[c]	0.02	0.006	0.007
	3-9	0.30	<0.001	<0.001
	3-10[c]	0.03	<0.001	<0.001
	3-11	0.34	<0.001	<0.001
	3-12	0.34	<0.001	<0.001

Supplemental Table 5. Comparisons of within-subject principal components (PCs) of phasor and Laguerre coefficient parameters using the *ex vivo* data based on univariable analysis with weighted linear mixed-effects models. Multi-leveled principal component analysis was used to construct principle components (PCs) for each Phasor or Laguerre Coefficient channel, with between-subject PCs (which reflect heterogeneity across animals) and within-subject PCs (which reflect variation within animals) to identify which spectral features to include in the multivariable model.

Within-subject principal component	Channel	Principal Component	Effect Size (Cohen's d)	P-value	Adjusted P-value
Phasor	1	PC1	-0.04	<0.001	<0.001
		PC2	-0.03	<0.001	<0.001
	2	PC1	-0.72	<0.001	<0.001

		PC2	-0.69	<0.001	<0.001
	3	PC1	-0.51	<0.001	<0.001
		PC2	-0.78	<0.001	<0.001
Laguerre Coefficient	1	PC1	-0.37	<0.001	<0.001
		PC2	0.46	<0.001	<0.001
	2	PC1	0.56	<0.001	<0.001
		PC2	-0.06	<0.001	<0.001
	3	PC1	0.49	<0.001	<0.001
		PC2	-0.06	<0.001	<0.001

Supplemental Table 6. Extraction of FLIm parameters across each spectral channel.

The sample fluorescence signal was expanded from the measured signal using a linear combination of Laguerre basis functions, where k is the discrete index of sampling.	$I(k)=\sum_{0}^{L-1} c_l b_l(k;\alpha)$
Fluorescence intensity in each spectral band was then determined by calculating the area under the curve of each decay	$I_k=\int_0^\infty i_k(t)dt$
Intensity ratios were then computed with the following formula	$IR_i=\frac{I_i}{\sum_{k=1}^{3} I_k}$
Average lifetimes were then computed with the following formula	$\langle\tau_k\rangle = \frac{\int_0^\infty t i_k(t)dt}{\int_0^\infty i_k(t)dt}$

Supplemental Table 7. **Description of the model hyper-parameters selection for decision tree model.**

Bayesian Optimization		
**Model**	**Hyperparameter**	**Range**
DT	Number of Estimators	50, 300
	Max Depth	3, 10
	Learning Rate	0.01, 0.3
	Gamma	0, 5
